# ONECUT2 facilitates hepatocellular carcinoma metastasis by transcriptionally upregulating FGF2 and ACLY

**DOI:** 10.1038/s41419-021-04410-3

**Published:** 2021-11-27

**Authors:** Danfei Liu, Tongyue Zhang, Xiaoping Chen, Bixiang Zhang, Yijun Wang, Meng Xie, Xiaoyu Ji, Mengyu Sun, Wenjie Huang, Limin Xia

**Affiliations:** 1 Department of Gastroenterology, Institute of Liver and Gastrointestinal Diseases, Hubei Key Laboratory of Hepato-Pancreato-Biliary Diseases, Tongji Hospital of Tongji Medical College, Huazhong University of Science and Technology, Wuhan, 430030 Hubei China; 2 Hubei Key Laboratory of Hepato-Pancreato-Biliary Diseases; Hepatic Surgery Center, Tongji Hospital, Tongji Medical College, Huazhong University of Science and Technology; Clinical Medicine Research Center for Hepatic Surgery of Hubei Province; Key Laboratory of Organ Transplantation, Ministry of Education and Ministry of Public Health, Wuhan, Hubei 430030 China

**Keywords:** Liver cancer, Metastasis

## Abstract

Metastasis is the predominant reason for high mortality of hepatocellular carcinoma (HCC) patients. It is critical to explore the molecular mechanism underlying HCC metastasis. Here, we reported that transcription factor One Cut homeobox 2 (ONECUT2) functioned as an oncogene to facilitate HCC metastasis. Elevated ONECUT2 expression was positively correlated with increased tumor number, tumor encapsulation loss, microvascular invasion, poor tumor differentiation, and advanced TNM stage. Mechanistically, ONECUT2 directly bound to the promoters of fibroblast growth factor 2 (FGF2) and ATP citrate lyase (ACLY) and transcriptionally upregulated their expression. Knockdown of FGF2 and ACLY inhibited ONECUT2-mediated HCC metastasis, whereas upregulation of FGF2 and ACLY rescued ONECUT2 knockdown-induced suppression of HCC metastasis. ONECUT2 expression was positively correlated with FGF2 and ACLY expression in human HCC tissues. HCC patients with positive coexpression of ONECUT2/FGF2 or ONECUT2/ACLY exhibited the worst prognosis. In addition, FGF2 upregulated ONECUT2 expression through the FGFR1/ERK/ELK1 pathway, which formed an FGF2-FGFR1-ONECUT2 positive feedback loop. Knockdown of ONECUT2 inhibited FGF2-induced HCC metastasis. Furthermore, the combination of FGFR1 inhibitor PD173074 with ACLY inhibitor ETC-1002 markedly suppressed ONECUT2-mediated HCC metastasis. In summary, ONECUT2 was a potential prognostic biomarker in HCC and targeting this oncogenic signaling pathway may provide an efficient therapeutic strategy against HCC metastasis.

## Introduction

Hepatocellular carcinoma (HCC) is a highly prevalent malignant disease worldwide, with the sixth highest diagnostic rate and the third highest mortality rate among cancers [1]. Although advances in surgery and chemotherapy have greatly improved the prognosis of HCC patients, those diagnosed with advanced HCC failed to receive effective treatments. Metastasis is the predominant cause of the low survival rate and ineffective treatments in HCC patients [2]. Elucidating the molecular characteristics of HCC is instrumental in developing potential therapeutic strategies. The aberrant expression of genes in HCC cells is likely involved in the occurrence and development of HCC. Expression profiles of clinical HCC samples have suggested that differentially expressed genes in HCC are regulated by liver-enriched transcription factors [3], stressing the importance of liver-enriched transcription factors in HCC. Therefore, identifying novel liver-enriched transcription factors is critical for a more comprehensive understanding of the molecular mechanism of HCC.

One cut homeobox 2 (ONECUT2), a liver-enriched transcription factor characterized by a single “cut” DNA-binding domain and an aberrant homeodomain, participates in liver differentiation and metabolism [4]. In addition, ONECUT2 regulates early liver expansion by controlling gene networks that affect cell adhesion and migration [5]. Interestingly, several studies reported that ONECUT2 expression is aberrantly upregulated in various types of solid tumors, such as colorectal cancer [6] and gastric cancer [7]. Overexpression of ONECUT2 facilitates cancer progression with roles in proliferation, invasion, and angiogenesis [8–10]. ONECUT2 is significantly elevated in HCC tissues and correlates with poor post-surgery survival in a cohort with 61 HCC patients [11]. However, its functional role in HCC remains largely unknown.

The aberrant activation of fibroblast growth factor (FGF)/FGF-receptor (FGFR) signaling accelerates HCC development through regulating angiogenesis, metabolism, and metastasis [12]. Fibroblast growth factor 2 (FGF2) (also known as bFGF), one of the 22 members of the FGF family, is ubiquitously expressed in epithelial cells, endothelial cells, fibroblasts, and macrophages [13]. FGF2 secreted by cancer cells regulates proliferation and motility of cancer cells by an autocrine mechanism and stimulates angiogenesis of endothelial cells by a paracrine mechanism, contributing to tumor progression [14]. FGF2 level is significantly elevated in HCC [15], and its level is closely correlated with HCC invasiveness [16].

The increase of de novo fatty acid synthesis is one of the most critical metabolic hallmarks of cancer cells, promoting tumorigenesis and tumor progression [17, 18]. ATP citrate lyase (ACLY) is the first rate-limiting enzyme that catalyses citrate to acetyl-CoA and oxaloacetate and then to fatty acids [19]. ACLY is significantly upregulated in several types of cancer including liver cancer [20], and facilitates the proliferation and metastasis of cancer cells [21, 22]. The expression of ACLY is regulated at the transcriptional and posttranslational levels, including transactivation [23], phosphorylation by AKT [24], acetylation by P300/calcium-binding protein-associated factor and deacetylation by sirtuin 2 [25], ubiquitination by Cullin 3-KLHL25 [26] and deubiquitination by ubiquitin-specific protease 30 [27].

In the present study, we demonstrated that ONECUT2 facilitated HCC metastasis by transactivating FGF2 and ACLY. FGF2, in turn, upregulated ONECUT2 expression through the FGFR1-extracellular signal-regulated protein kinase (ERK)-ELK1 signaling pathway, thus forming an FGF2/FGFR1/ONECUT2 positive feedback loop. The FGFR1 inhibitor PD173074 combined with the ACLY inhibitor ETC-1002 markedly suppressed ONECUT2-induced HCC metastasis.

## Results

### ONECUT2 is overexpressed in HCC tissues and accelerates HCC metastasis

**Fig. 1:**
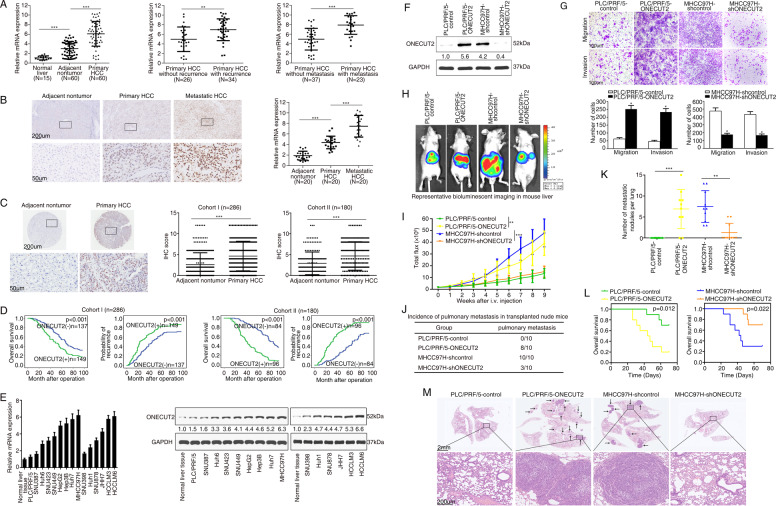
ONECUT2 is overexpressed in HCC tissues and accelerates HCC metastasis. **A** Left: *ONECUT2* mRNA levels were detected in 15 normal liver tissues and 60 pairs of HCC and adjacent nontumor tissues by RT-PCR. Middle: *ONECUT2* mRNA levels in HCC samples from patients with recurrence (*n* = 34) or without recurrence (*n* = 26). Right: *ONECUT2* mRNA levels in HCC samples from patients with metastasis (*n* = 23) or without metastasis (*n* = 37). **B** Representative IHC staining (left) and RT-PCR (right) showing ONECUT2 levels in 20 pairs of fresh metastatic and matched primary HCC tissues and adjacent nontumor tissues. **C** Representative IHC staining (left) and IHC scores (right) showing ONECUT2 levels in two HCC cohorts. **D** Prognosis of HCC patients was compared between negative and positive ONECUT2 expression group by Kaplan–Meier method in two HCC cohorts. **E** RT-PCR (left) and western blotting (right) showing ONECUT2 levels in normal liver tissues and HCC cells. **F** Protein levels of ONECUT2 were measured by western blotting when PLC/PRF/5 cells were transfected with ONECUT2-overexpressing lentivirus and when MHCC97H cells were transfected with ONECUT2-silencing lentivirus. **G** Transwell assays were used to evaluate migration and invasion of ONECUT2-overexpressing PLC/PRF/5 cells and ONECUT2-silencing MHCC97H cells. ONECUT2 overexpression promoted HCC metastasis. Representative bioluminescent imaging (**H**), bioluminescent signals (**I**), the incidence of pulmonary metastasis (**J**), number of pulmonary metastasis foci (**K**), overall survival (**L**), and H&E staining of the lungs (**M**) among four groups with ten mice in each group 9 weeks after orthotopic implantation of the indicated cells. Data are mean ± SD. *P < 0.05, **P < 0.01, ***P < 0.001.

First, we analysed ONECUT2 expression from The Cancer Genome Atlas Liver Hepatocellular Carcinoma (TCGA-LIHC; http://timer.cistrome.org/). The result showed the overexpression of ONECUT2 in HCC tissues (Supplementary Fig. S1A). Next, ONECUT2 expression was examined in human normal liver tissues (*n* = 15), and paired primary HCC and adjacent nontumor tissues (*n* = 60) by real-time PCR (RT-PCR). Primary HCC tissues had the highest mRNA levels of *ONECUT2*, followed by adjacent nontumor tissues, and finally normal liver tissues. Primary HCC patients with recurrence (34 of 60) and metastasis (23 of 60) exhibited higher levels of ONECUT2 than those without recurrence (26 of 60) or metastasis (37 of 60) (Fig. 1A). Additionally, higher ONECUT2 expression was detected in metastatic HCC tissues than in primary HCC tissues and adjacent nontumor tissues (Fig. 1B). The upregulation of ONECUT2 in metastatic HCC tissues was validated by western blotting (Supplementary Fig. S1B). We further evaluated the levels and clinicopathological relevance of ONECUT2 in a 286-patient cohort (Cohort I) and a 180-patient cohort (Cohort II). A strong positive staining pattern for ONECUT2 was observed in HCC tissues (Fig. 1C). HCC patients who were ONECUT2-positive had a poorer prognosis than those who were ONECUT2-negative (Fig. 1D). Positive ONECUT2 expression was significantly correlated with increased tumor number, tumor encapsulation loss, microvascular invasion, poor tumor differentiation, and advanced tumor-node-metastasis (TNM) stage (Table 1) and was predictive of a poor outcome in both HCC cohorts (Supplementary Tables S1 and S2).

**Table 1:** Correlation between ONECUT2 expression and clinicopathological characteristics of HCCs in two independent cohorts of human HCC tissues.

		Cohort I			Cohort II		
Clinicopathological variables		Tumor ONECUT2 expression		*P* value	Tumor ONECUT2 expression		*P* value
		Negative (*n* = 137)	Positive (*n* = 149)		Negative (*n* = 84)	Positive (*n* = 96)	
Age		51.77 (9.160)	53.36 (8.559)	0.301	50.46 (10.155)	51.64 (10.53)	0.882
Sex	Female	18	29	0.155	14	18	0.845
	Male	119	120		70	78	
Serum AFP	≤20 ng/ml	51	44	0.209	15	27	0.115
	>20 ng/ml	86	105		69	69	
Virus infection	HBV	101	103	0.710	65	68	0.431
	HCV	11	18		9	8	
	HBV + HCV	7	8		2	5	
	None	18	20		8	15	
Cirrrhosis	Absent	41	56	0.211	27	24	0.322
	Present	96	93		57	72	
Child-pugh score	Class A	116	135	0.150	65	72	0.730
	Class B	21	14		19	24	
Tumor number	Single	76	63	0.033*	46	29	0.001*
	Multiple	61	86		38	67	
Maximal tumor size	≤5 cm	91	77	0.012*	44	36	0.052
	>5 cm	46	72		40	60	
Tumor encapsulation	Absent	33	54	0.029*	25	57	<0.001*
	Present	104	95		59	39	
Microvascular invasion	Absent	88	70	0.004*	63	38	<0.001*
	Present	49	79		21	58	
Tumor differentiation	I–II	102	92	0.023*	67	49	<0.001*
	III–IV	35	57		17	47	
TNM stage	I–II	116	96	<0.001*	80	63	<0.001*
	III	21	53		4	33	

Next, we detected the levels of ONECUT2 in HCC cells with different metastatic potentials. Highly metastatic HCC cells expressed higher ONECUT2 levels than those with low metastatic capacity (Fig. 1E). PLC/PRF/5 cells were transfected with ONECUT2-overexpressing lentivirus to establish PLC/PRF/5-ONECUT2 cell lines (Fig. 1F). Three different short hairpin RNAs (shRNAs) targeting ONECUT2 were designed to silence ONECUT2 expression. MHCC97H cells transfected with shONECUT2-1 lentivirus showed the least expression of ONECUT2 and the weakest capabilities of migration and invasion (Supplementary Fig. S1C–D); thus, these cells were selected for further experiments. ONECUT2 overexpression promoted HCC proliferation, migration and invasion, while ONECUT2 knockdown exhibited the opposite effect (Fig. 1G and Supplementary Fig. S1E, F). Mice with liver orthotopic tumor implantation of PLC/PRF/5-ONECUT2 cells exhibited faster tumor growth, more pulmonary metastasis, and worse overall survival. In contrast, attenuated tumor growth, decreased pulmonary metastasis, and improved overall survival were observed in mice with liver orthotopic tumor implantation of MHCC97H-shONECUT2 cells (Fig. 1H–M). These results indicated that ONECUT2 overexpression promoted HCC metastasis.

### FGF2 and ACLY are two downstream molecules of ONECUT2

**Fig. 2:**
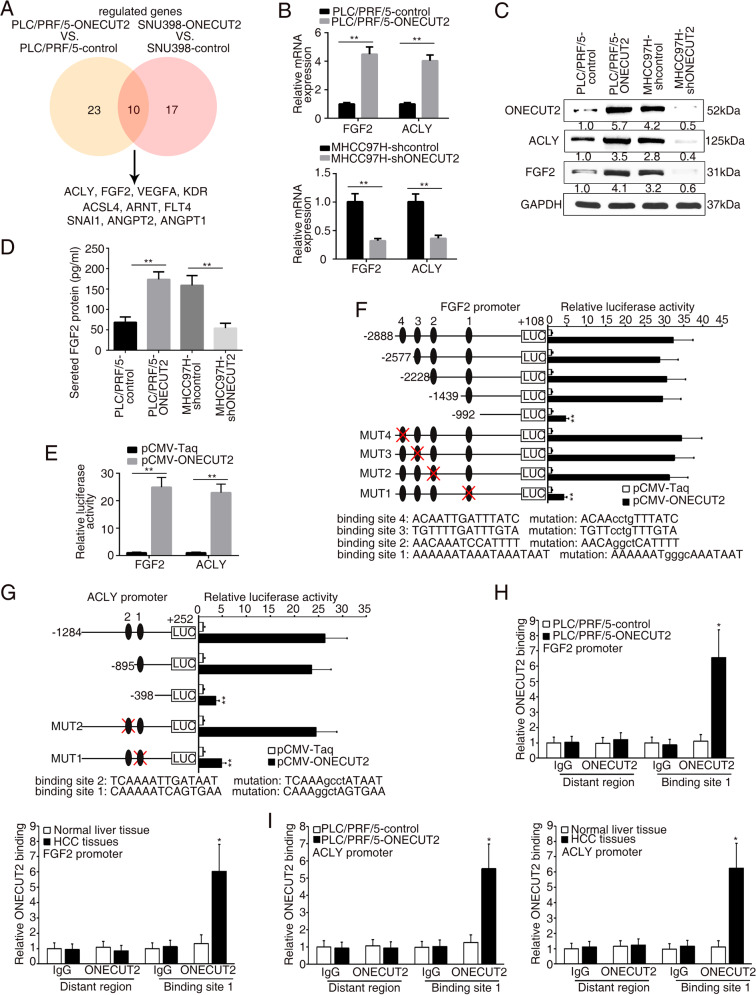
FGF2 and ACLY are two downstream molecules of ONECUT2. **A** The human Cancer PathwayFinder RT^2^ Profiler PCR Array was performed in PLC/PRF/5 and SNU398 cells infected with ONECUT2-overexpressing lentivirus. Venn diagram showing the overlap of upregulated genes in both PLC/PRF/5-ONECUT2 and SNU398-ONECUT2 cells (fold change >2.0). **B** RT-PCR showing mRNA levels of *FGF2* and *ACLY* in ONECUT2-overexpressing PLC/PRF/5 cells and ONECUT2-silencing MHCC97H cells. **C** Protein levels of FGF2 and ACLY were measured in ONECUT2-overexpressing PLC/PRF/5 cells and ONECUT2-silencing MHCC97H cells by western blotting. **D** The level of secreted FGF2 was measured in ONECUT2-overexpressing PLC/PRF/5 cells and ONECUT2-silencing MHCC97H cells by ELISA. **E** Luciferase reporter assays were performed in PLC/PRF/5 cells after cotransfection with *FGF2* or *ACLY* promoter and pCMV-ONECUT2 plasmid. **F**–**G** The relative luciferase activities were measured in PLC/PRF/5 cells after cotransfection with truncations and mutations of *FGF2* or *ACLY* promoter and pCMV-ONECUT2 plasmid. **H**–**I** The direct binding of ONECUT2 to the promoters of *FGF2* and *ACLY* was determined in HCC cells and tissues by ChIP assays. Data are mean ± SD. *P < 0.05, **P < 0.01.

To identify the molecular mechanism involved in ONECUT2-induced HCC metastasis, ONECUT2-overexpressing lentivirus was transfected into PLC/PRF/5 and SNU398 cells, subsequently the human Cancer PathwayFinder RT^2^ Profiler PCR Array was conducted. In total, 23 genes in PLC/PRF/5 cells and 17 genes in SNU398 cells showed 2-fold changes in expression following ONECUT2 overexpression, respectively (Supplementary Tables S3 and S4). By intersecting the above two dataset, we acquired ten overlapped genes including *ACLY*, *FGF2*, *VEGFA*, *KDR*, *ACSL4*, *ARNT*, *FLT4*, *SNAI1*, *ANGPT2*, and *ANGPT1* (Fig. 2A). *ACLY* and *FGF2* were the potential targets of ONECUT2 with the highest fold change. TCGA-LIHC database also showed a significant correlation between *ONECUT2* expression and *FGF2* and *ACLY* expression (Supplementary Fig. S2; http://gepia.cancer-pku.cn/; http://timer.cistrome.org/). RT-PCR and western blotting confirmed that ONECUT2 upregulation significantly promoted FGF2 and ACLY expression, while ONECUT2 downregulation inhibited FGF2 and ACLY expression (Fig. 2B, C). Enzyme-linked immunosorbent assay (ELISA) showed that ONECUT2 overexpression increased FGF2 secretion, while ONECUT2 silence decreased FGF2 secretion (Fig. 2D). Importantly, the promoter activities of *FGF2* and *ACLY* were significantly enhanced by ONECUT2 overexpression (Fig. 2E).

Several putative ONECUT2 binding motifs were identified in the promoters of *FGF2* and *ACLY* by sequence analysis (Supplementary Figs. S3 and S4). We designed a series of deletions and mutations of *FGF2* and *ACLY* promoter. The ONECUT2-enhanced activity of *FGF2* promoter was significantly attenuated by deleting the region between −1439 and −992 bp and mutating binding site 1 in the *FGF2* promoter (Fig. 2F). Similarly, deleting the region between −895 and −398 bp or mutating binding site 1 in the *ACLY* promoter significantly inhibited reporter activity induced by ONECUT2 overexpression (Fig. 2G). Chromatin immunoprecipitation (ChIP) assays determined the direct interaction between ONECUT2 and the promoters of *FGF2* and *ACLY* in HCC cells and tissues (Fig. 2H, I). These results indicated that ONECUT2 transactivated FGF2 and ACLY expression.

### ONECUT2 levels are positively correlated with FGF2 and ACLY levels in HCC tissues

**Fig. 3:**
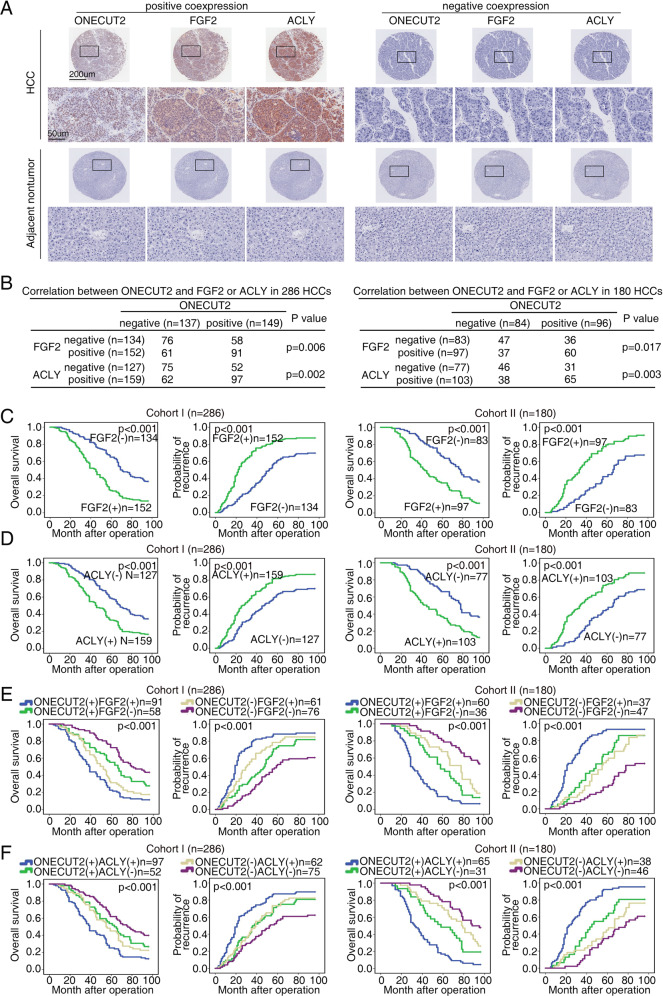
ONECUT2 levels are positively correlated with FGF2 and ACLY levels in HCC tissues. **A** Representative IHC staining for ONECUT2, FGF2, and ACLY in HCC and adjacent nontumor tissues. **B** Correlation analyses of protein expression between ONECUT2 and ACLY or FGF2 in two HCC cohorts. **C**, **D** Prognosis of HCC patients was compared between negative and positive FGF2 expression group and between negative and positive ACLY expression group by Kaplan–Meier method in two HCC cohorts. **E**, **F** Prognosis of HCC patients was compared based on the coexpression of ONECUT2 and ACLY or FGF2 by Kaplan–Meier method in two HCC cohorts.

The possible correlation between ONECUT2 expression and FGF2 or ACLY expression was further evaluated in two HCC cohorts. A positive correlation of ONECUT2 expression with FGF2 or ACLY expression was determined by immunohistochemistry (IHC) staining (Fig. 3A, B). Overexpression of FGF2 and ACLY correlated with malignant characteristics (Supplementary Table S5 and S6). HCC patients who were ACLY- or FGF2-positive exhibited worse prognosis than those who were ACLY- or FGF2-negative (Fig. 3C, D). Furthermore, HCC patients with ONECUT2/FGF2 coexpression or ONECUT2/ACLY coexpression exhibited the worst prognosis (Fig. 3E, F).

### ONECUT2 promotes HCC metastasis by upregulating FGF2 and ACLY

**Fig. 4:**
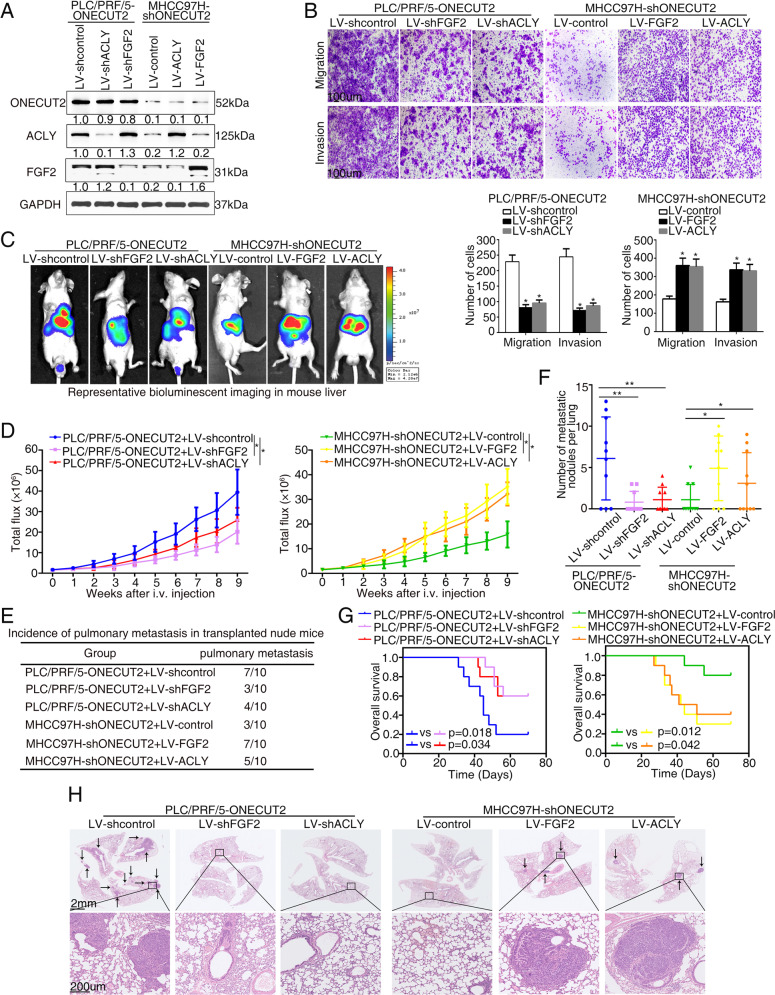
ONECUT2 promotes HCC metastasis by upregulating FGF2 and ACLY. **A** Protein levels of ONECUT2, FGF2, and ACLY were measured in PLC/PRF/5 cells cotransfected with ONECUT2-overexpressing lentivirus and FGF2-silencing or ACLY-silencing lentivirus and MHCC97H cells cotransfected with ONECUT2-silencing lentivirus and FGF2-overexpressing or ACLY-overexpressing lentivirus by western blotting. **B** Knockdown of FGF2 or ACLY was performed in ONECUT2-overexpressing PLC/PRF/5 cells, and overexpression of FGF2 or ACLY was performed in ONECUT2-silencing MHCC97H cells. Next, transwell assays were performed. **C**–**H** ONECUT2 facilitated HCC metastasis by inducing FGF2 and ACLY. Representative bioluminescent imaging (**C**), bioluminescent signals (**D**), the incidence of pulmonary metastasis (**E**), number of pulmonary metastasis foci (**F**), overall survival (**G**), and H&E staining of the lungs (**H**) among four groups with ten mice in each group 9 weeks after orthotopic implantation of the indicated cells. Data are mean ± SD. *P < 0.05, **P < 0.01.

To verify the participation of FGF2 and ACLY in ONECUT2-mediated HCC metastasis, we knocked down FGF2 and ACLY in ONECUT2-overexpressing PLC/PRF/5 cells, and upregulated FGF2 and ACLY in ONECUT2-silencing MHCC97H cells (Supplementary Fig. S5 and Fig. 4A). FGF2 or ACLY downregulation attenuated ONECUT2-mediated migration and invasion of PLC/PRF/5 cells, while FGF2 or ACLY upregulation rescued the decrease in migration and invasion of MHCC97H cells induced by ONECUT2 knockdown (Fig. 4B). In vivo metastasis assay showed that FGF2 or ACLY inhibition significantly decreased pulmonary metastasis and increased overall survival in mice xenografted with PLC/PRF/5-ONECUT2 cells. In contrast, FGF2 or ACLY overexpression markedly enhanced pulmonary metastasis and decreased overall survival in MHCC97H-shONECUT2 xenograft mice (Fig. 4C–H). These findings indicated that ONECUT2 facilitated HCC metastasis by upregulating FGF2 and ACLY.

### FGF2 upregulates ONECUT2 expression through the FGFR1/ERK/ELK1 signaling pathway

**Fig. 5:**
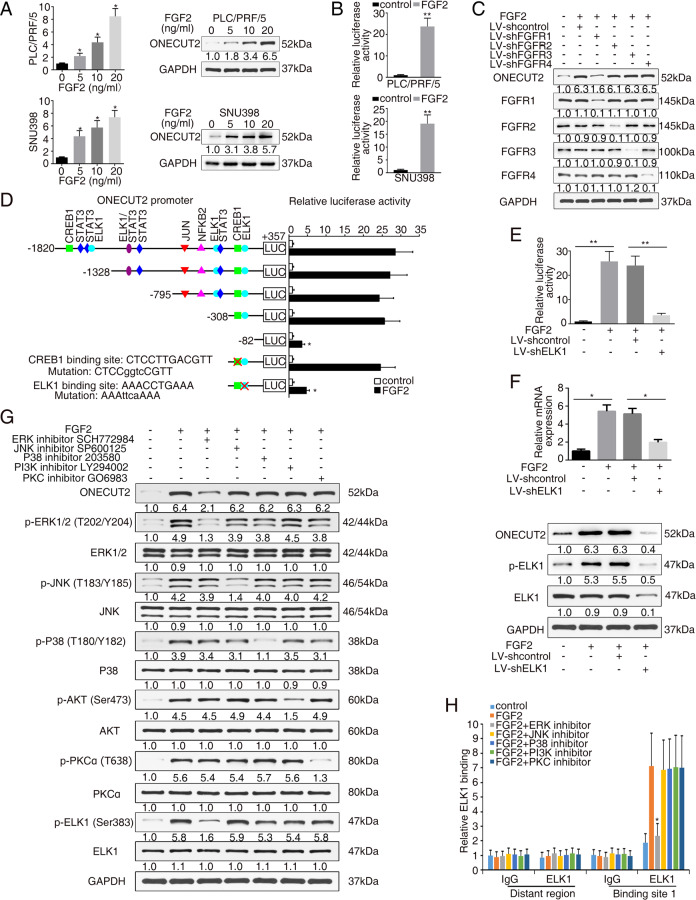
FGF2 upregulates ONECUT2 expression through the FGFR1/ERK/ELK1 signaling pathway. **A** The levels of ONECUT2 were detected by RT-PCR and western blotting after treatment with different concentrations of FGF2 (0, 5, 10, 20 ng/ml) for 24 h. **B** Luciferase reporter assay showing the promoter activity of *ONECUT2* in PLC/PRF/5 cells and SNU398 cells after FGF2 treatment (20 ng/ml, 24 h). **C** The levels of ONECUT2 were detected in PLC/PRF/5 cells after FGF2 treatment (20 ng/ml, 24 h) and FGFR1, FGFR2, FGFR3, or FGFR4 knockdown by western blotting. **D** The relative luciferase activity was detected in PLC/PRF/5 cells after transfection with truncations and mutated *ONECUT2* promoter constructs, followed by FGF2 treatment (20 ng/ml, 24 h). **E** HCC cells were transfected with ELK1-silencing lentivirus, followed by FGF2 treatment (20 ng/ml, 24 h). Luciferase reporter assay showing *ONECUT2* promoter activity. **F** RT-PCR and western blotting showing ONECUT2 levels in HCC cells transfected with ELK1-silencing lentivirus followed by FGF2 treatment (20 ng/ml, 24 h). **G** Protein levels of ONECUT2, ERK, p-ERK, JNK, p-JNK, P38, p-P38, AKT, p-AKT, PKCα, and p-PKCα were measured by western blotting when PLC/PRF/5 cells were pretreated with inhibitors of ERK (SCH772984, 10 μM, 30 min), JNK (SP600125, 20 μM, 1 h), P38 (SB203580, 20 μM, 1 h), PI3K (LY294002, 20 μM, 1 h) or PKC (GO6983, 10 μM, 30 min), followed by administration of FGF2 (20 ng/ml, 24 h). **H** Relative binding of ELK1 to *ONECUT2* promoter was determined by ChIP assays when HCC cells were treated with FGF2 (20 ng/ml, 24 h) and the indicated inhibitor. Data are mean ± SD. *P < 0.05, **P < 0.01.

FGFs are involved in several cellular processes of tumor progression, such as stemness, proliferation, antiapoptosis, migration, and angiogenesis, by binding with their receptors [28]. FGF2-producing HCC cells release FGF2 into conditioned media to transfer growth signals [29]. We questioned whether FGF2 could in turn regulate ONECUT2 expression. After treatment with different concentrations of FGF2 for 24 h, ONECUT2 expression showed a dose-dependent upregulation (Fig. 5A). Interestingly, the promoter activity of *ONECUT2* was markedly enhanced following FGF2 treatment (Fig. 5B), suggesting that FGF2 could transactivate ONECUT2 expression. FGF signals are transduced through FGF receptors (FGFRs). To determine which FGFR was responsible for FGF2-stimulated ONECUT2 expression, FGFR1, FGFR2, FGFR3, and FGFR4 were separately knocked down in PLC/PRF/5 cells. FGF2-stimulated ONECUT2 expression was significantly blocked following FGFR1 knockdown (Fig. 5C), indicating that FGF2-FGFR1 signaling was involved in the upregulation of ONECUT2 expression in HCC.

To identify the cis-regulatory region, we analysed the promoter sequence of *ONECUT2* by NCBI and identified several potential transcription factor binding sites (Supplementary Fig. S6). Then, we constructed luciferase reporter plasmids containing truncations and mutations of the *ONECUT2* promoter. The promoter activity of *ONECUT2* markedly decreased when the region from −308 to −82 bp was truncated (Fig. 5D), suggesting that this region was crucial for FGF2-induced ONECUT2 activation. The potential binding motifs of cAMP responsive element binding protein 1 (CREB1) and ELK1 are located in this region. Upon mutating the binding motif of ELK1, the luciferase activity significantly decreased, indicating that the binding of ELK1 to the promoter of *ONECUT2* was crucial for FGF2-induced ONECUT2 activation (Fig. 5D). Importantly, ELK1 knockdown significantly impaired the FGF2-enhanced promoter activity and expression of ONECUT2 (Fig. 5E, F). Several intracellular pathways can be activated by FGF-FGFR signaling, including the Ras-mitogen-activated protein kinase, phosphoinositide 3-kinase (PI3K)-Akt, and PLC gamma (PLCγ) -Ca^2+^-protein kinase C (PKC) pathways [30]. To identify the pathway involved in FGF2-induced ONECUT2 expression, specific inhibitors targeting these signaling pathways were administered to PLC/PRF/5 cells, including an ERK inhibitor (SCH772984), a c-Jun N-terminal kinase (JNK) inhibitor (SP600125), a P38 inhibitor (SB203580), a PI3K inhibitor (LY294002) and a PKC inhibitor (GO6983). ONECUT2 upregulation and ELK1 activation induced by FGF2 were significantly blocked by the ERK inhibitor (Fig. 5G). Furthermore, the ERK inhibitor, but not the other inhibitors, prevented ELK1 from binding to the *ONECUT2* promoter (Fig. 5H). Together, these data suggested that FGF2 promoted ONECUT2 expression via the ERK/ELK1 pathway.

### ONECUT2 is crucial for FGF2-induced HCC metastasis

**Fig. 6:**
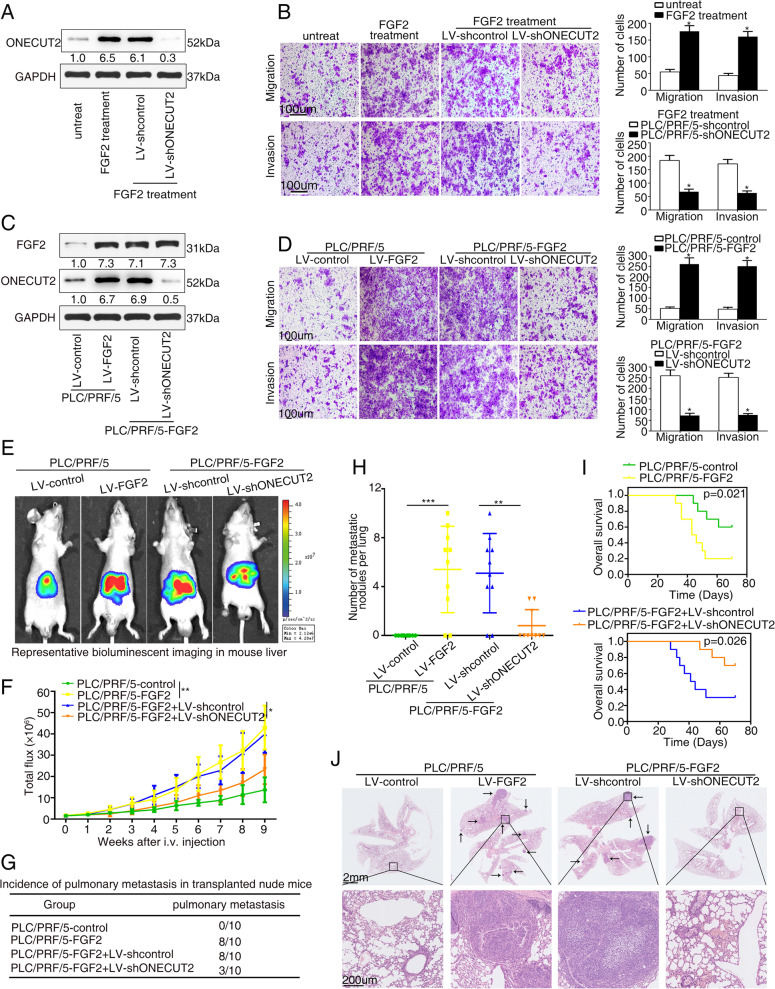
ONECUT2 is crucial for FGF2-induced HCC metastasis. **A** Protein levels of ONECUT2 were measured in PLC/PRF/5 cells after ONECUT2 knockdown and FGF2 treatment (20 ng/ml, 24 h) by western blotting. **B** Transwell assays were used to evaluate migration and invasion of PLC/PRF/5 cells after ONECUT2 knockdown and FGF2 treatment (20 ng/ml, 24 h). **C** Protein levels of FGF2 and ONECUT2 were measured in PLC/PRF/5 cells after FGF2 overexpression and ONECUT2 knockdown by western blotting. **D** Transwell assays were used to evaluate migration and invasion of PLC/PRF/5 cells after FGF2 overexpression and ONECUT2 knockdown. **E**–**J** ONECUT2 knockdown inhibited FGF2-enhanced HCC metastasis. Representative bioluminescent imaging (**E**), bioluminescent signals (**F**), the incidence of pulmonary metastasis (**G**), number of pulmonary metastasis foci (**H**), overall survival (**I**), and H&E staining of the lungs (**J**) among four groups with ten mice in each group 9 weeks after orthotopic implantation of the indicated cells. Data are mean ± SD. *P < 0.05, **P < 0.01.

The regulation of ONECUT2 by FGF2 prompted us to explore the role of ONECUT2 in FGF2-mediated HCC metastasis. We knocked down ONECUT2 by lentivirus in PLC/PRF/5 cells and treated them with FGF2 (Fig. 6A). FGF2-stimulated PLC/PRF/5 cell migration and invasion, but this effect was blocked by ONECUT2 knockdown (Fig. 6B). We further established PLC/PRF/5-FGF2 cells and MHCC97H-shFGF2 cells (Fig. 6C and Supplementary Fig. 7C) and found that ONECUT2 knockdown attenuated the proliferation, migration and invasion of PLC/PRF/5-FGF2 cells (Fig. 6D and Supplementary Fig. 7A, B). In contrast, ONECUT2 overexpression rescued the reduction of proliferation, migration and invasion of MHCC97H-shFGF2 cells (Supplementary Fig. 7D–F). Consistent with these findings, FGF2 overexpression accelerated tumor growth, enhanced pulmonary metastasis, and decreased overall survival, which were reversed by ONECUT2 knockdown (Fig. 6E–J). The above data indicated that ONECUT2 was indispensable for FGF2-mediated HCC metastasis.

### PD173074 in combination with ETC-1002 significantly suppresses ONECUT2-mediated HCC metastasis

**Fig. 7:**
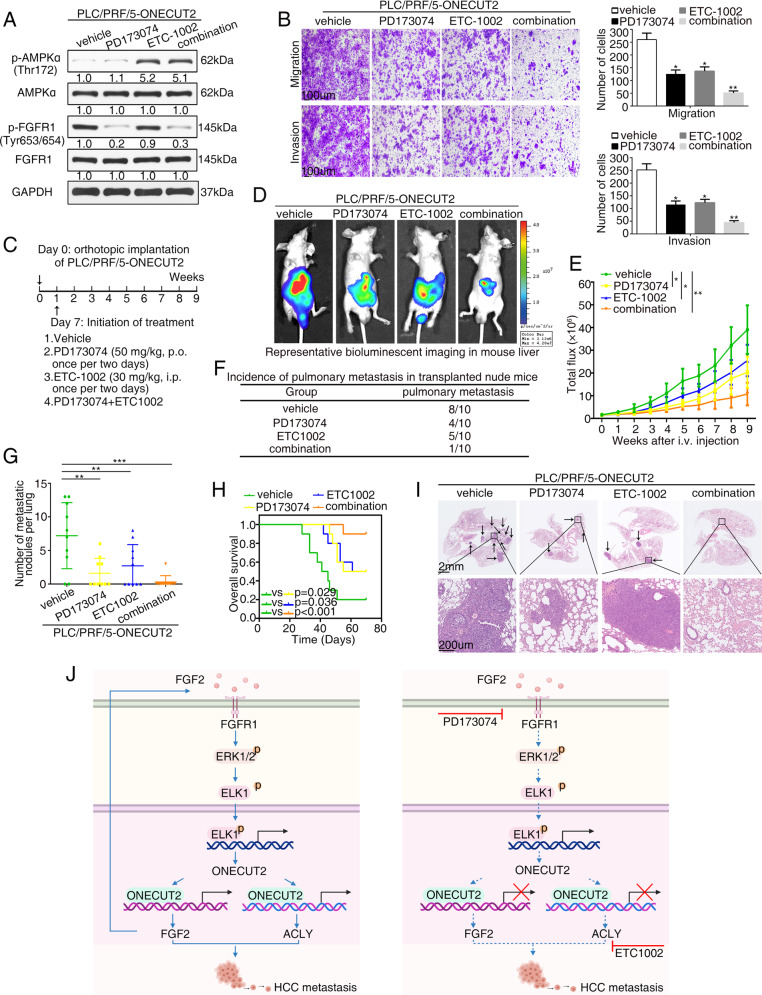
PD173074 in combination with ETC-1002 significantly suppresses ONECUT2-mediated HCC metastasis. **A** Protein levels of p-AMPKα, AMPKα, p-FGFR1, and FGFR1 were measured in ONECUT2-overexpressing PLC/PRF/5 cells after treatment with PD173074 (5 μM, 12 h), ETC-1002 (100 μM, 12 h) or PD173074 combined with ETC-1002 by western blotting. **B** Transwell assays were used to evaluate migration and invasion of the indicated cells. **C** Diagram of the administration strategies in vivo. One week after the orthotopic implantation, the nude mice were administered PD173074 (50 mg/kg), ETC-1002 (30 mg/kg) or PD173074 combined with ETC-1002. **D**–**I** PD173074 combined with ETC-1002 markedly inhibited ONECUT2-enhanced HCC metastasis. Representative bioluminescent imaging (**D**), bioluminescent signals (**E**), the incidence of pulmonary metastasis (**F**), number of pulmonary metastasis foci (**G**), overall survival (**H**), and H&E staining of the lungs (**I**) among four groups of nude mice treated with different administration strategies (*n* = 10 in each group) for 8 weeks following orthotopic implantation of PLC/PRF/5-ONECUT2 cells. Data are mean ± SD. *P < 0.05, **P < 0.01, ***P < 0.001. **J** Proposed model showing the role of ONECUT2 in facilitating HCC metastasis. FGF2 upregulates ONECUT2 expression via FGFR1/ERK/ELK1 signaling pathway. ONECUT2 facilitates HCC metastasis through transcriptionally upregulating FGF2 and ACLY expression. The combination of FGFR1 inhibitor with ACLY inhibitor dramatically suppresses ONECUT2-mediated HCC metastasis.

Based on the finding that FGF2 and ACLY collaboratively mediated the carcinogenic effect of ONECUT2, we investigated the efficacy of a combined intervention targeting FGF2 and ACLY against ONECUT2-mediated HCC metastasis. The cancer-promoting activity of FGF2 can be blocked by various strategies, including ligand-binding inhibitors, tyrosine kinase inhibitors, and anti-FGFR antibodies [31]. PD173074, a potent FGFR1 inhibitor, exhibits apparent antitumor activity in basal-like breast cancer cell lines that express autocrine FGF2 [32]. ETC-1002, an ACLY inhibitor and adenosine monophosphate-activated protein kinase (AMPK) activator, has been applied as a cholesterol-reducing agent in patients with hypercholesterolemia [33]. Here, PLC/PRF/5-ONECUT2 cells were incubated with PD173074, ETC-1002, or both agents. PD173074 significantly decreased FGFR1 phosphorylation, and ETC-1002 strongly increased AMPK phosphorylation (Fig. 7A). PD173074 combined with ETC-1002 was more potent in inhibiting PLC/PRF/5-ONECUT2 cell migration and invasion than PD173074 or ETC-1002 alone (Fig. 7B). Furthermore, PD173074, ETC-1002 or PD173074 combined with ETC-1002 was administered to xenograft nude mice (Fig. 7C). Treatment was initiated 1 week after the orthotopic implantation of PLC/PRF/5-ONECUT2 cells and lasted 8 weeks. PD173074 (50 mg/kg) was administered orally once every 2 days, and ETC-1002 (30 mg/kg) was administered intravenously once every 2 days. The combined administration of PD173074 and ETC-1002 significantly lowered pulmonary metastasis and improved overall survival in PLC/PRF/5-ONECUT2 xenograft mice (Fig. 7D–I). These findings indicated that combined treatment with PD173074 and ETC-1002 more effectively suppressed ONECUT2-induced HCC metastasis.

## Discussion

The development of various tissues and organs results from the coordinated synthesis of a set of tissue-specific proteins in time and space. Transcription factors play a crucial role in this process. For example, genes essential for hepatocytic cell lineage are transcriptionally regulated by hepatocyte nuclear factors [34]. Dysfunction of transcription factors is closely associated with several human diseases, including cancers [35]. Liver-enriched transcription factors are essential for liver function and development under physiological conditions [36]. However, the role of dysregulated liver-enriched transcription factors in HCC is little known. Here, we found that ONECUT2, a liver-enriched transcription factor, was overexpressed in HCC tissues, particularly in metastatic HCC tissues, and facilitated HCC metastasis. Positive ONECUT2 expression was statistically related to an increased tumor number, tumor encapsulation loss, microvascular invasion, poor tumor differentiation, and advanced TNM stage. Patients who were ONECUT2-positive exhibited a poor prognosis. However, the mechanism of ONECUT2 involved in HCC metastasis needs to be further studied.

To identify the regulatory pathways involved in ONECUT2-mediated HCC metastasis, we conducted a human Cancer PathwayFinder RT^2^ Profiler PCR Array following ONECUT2 overexpression. The results indicated that *FGF2* and *ACLY* were the two most significantly upregulated genes. Previous studies demonstrated that FGF2 induces FGFR1 phosphorylation and activates downstream ERK signaling in HCC cells [37]. ERK signaling accelerates epithelial-mesenchymal transition process in HCC cells by modulating matrix metalloproteinases (MMPs) expression [38]. ACLY is the major source of acetyl-CoA for histone acetylation [39]. Increased acetyl-CoA levels induce H3 acetylation in the promoter region of *TWIST2* and promote HCC metastasis [40]. In our study, FGF2 and ACLY exhibited a synergetic effect in ONECUT2-mediated HCC metastasis. Both knockdown and pharmacological intervention of FGF2 or ACLY attenuated ONECUT2-mediated HCC metastasis. Simultaneously targeting FGF2 and ACLY further suppressed ONECUT2-mediated HCC metastasis.

However, the mechanism for ONECUT2 upregulation in HCC is still unknown. FGF signaling plays a role in inducing liver-specific gene expression [41]. Our data demonstrated that FGF2 regulated ONECUT2 expression by binding with its receptor FGFR1 and activating ERK/ELK1 signaling, forming an FGF2/FGFR1/ONECUT2 positive feedback loop. Thus, FGF2 was the upstream regulator of ACLY through the regulation of ONECUT2. We speculated that ACLY may play an important part in FGF2-mediated HCC proliferation and metastasis. To verify this hypothesis, we knocked down ACLY in FGF2-overexpressing PLC/PRF/5 cells (Supplementary Fig. S8A). The results showed that ACLY knockdown could inhibit FGF2-mediated cell proliferation, migration and invasion (Supplementary Fig. S8B–D). Normal hepatocytes do not express FGFR1, but FGFR1 is detectable in HCC cells [42]. This finding indicates that liver tissues start to express FGFR1 at high levels in tumorgenesis, and increased FGFR1 expression may be involved in HCC progression. Targeting FGFR1 is a strategy to block FGF2-induced HCC progression. In our study, FGFR1 knockdown blocked FGF2-stimulated ONECUT2 expression, thus disrupting this loop.

Therapies for advanced HCC with vascular invasion and extrahepatic spread are limited and unsatisfactory. A thorough biological understanding of HCC metastasis is the basis for developing treatments for metastatic HCC. HCC metastasis is a multi-step and multi-stage process. First, cancer cells must escape their primary sites and enter the blood circulation. Next, the cells migrate to new sites, where they rapidly proliferate to form macroscopic metastases [43]. Angiogenesis and lipid metabolism respectively provide escape routes and metabolic intermediates, which are critical for the development of invasive HCC [44, 45]. Angiogenesis and lipid metabolism play a synergistic role in tumorigenesis. On the one hand, angiogenesis provides a blood supply for metabolic needs during tumorigenesis [46]. On the other hand, cancer lipid metabolism confers antiangiogenic drug resistance [47]. Therefore, targeting angiogenesis and lipid metabolism could be exploited for the treatment of metastatic HCC. In our study, ONECUT2 overexpression promoted HCC metastasis by transactivating FGF2 and ACLY expression. Simultaneous blockade of FGF2 and ACLY may inhibit ONECUT2-mediated HCC metastasis. PD173074 is a tyrosine kinase and angiogenesis inhibitor targeting FGFR1 and VEGFR2. PD173074 effectively blocks angiogenesis induced by FGF2 or VEGFA with no apparent toxicity in mice [48]. The liver-specific inhibition of ETC-1002 on ACLY prevents hepatic steatosis, attracting interest as a potential therapeutic strategy for HCC [49]. We found that both PD173074 and ETC-1002 inhibited ONECUT2-mediated HCC metastasis when used alone, but the inhibitory effect was more obvious when used in combination, providing a potential therapeutic strategy for ONECUT2-driven HCC metastasis.

In conclusion, ONECUT2 promoted HCC metastasis by transactivating FGF2 and ACLY expression. FGF2-FGFR1 signaling, in turn, upregulated ONECUT2 expression through ERK/ELK1 axis, thus forming a positive feedback loop. Considering the universal occurrence of resistance to single agents and the more obvious suppressive effect of combination therapy in HCC metastasis, the combined administration of PD173074 and ETC-1002 may be a promising strategy for HCC treatment (Fig. 7J).

## Materials and methods

### Cell culture and reagents

Huh1, Huh6, SNU878, JHH-7 and Huh7 cells were obtained from the Institute of Biochemistry and Cell Biology, Chinese Academy of Science, China. HepG2, Hep3B, PLC/PRF/5, SNU387, SNU423, SNU398, and SNU449 cells were purchased from ATCC (Manassas, VA, USA). MHCC97H, HCCLM3, and HCCLM6 cells were kindly provided by Dr. Tang ZY (Liver Cancer Institute, Zhongshan Hospital, Fudan University, Shanghai, China). Cells were cultured in DMEM or 1640 medium with 10% FBS, 100 μg/ml penicillin, and 100 μg/ml streptomycin at 37 °C in a 5% CO2 incubator. All the cell lines were certified by the STR.

The ERK inhibitor SCH772984 (HY-50846), JNK inhibitor SP600125 (HY-12041), P38 inhibitor SB203580 (HY-10256), PI3K inhibitor LY294002 (HY-10108), PKC inhibitor GO6983 (HY-13689), recombinant human basic FGF (HY-P7004), FGFR1 inhibitor PD173074 (HY10321) and ACLY inhibitor ETC-1002 (HY-12357) were purchased from MedChemExpress.

### Patients and follow-up

This study enrolled 286 and 180 adult patients with HCC who underwent surgical resection at the Tongji Hospital of Tongji Medical College (Wuhan, China) between 2003 and 2005 and between 2006 and 2008 respectively. Tumor staging was based on the AJCC/UICC TNM classification of HCC. 15 normal liver tissues, 60 paired fresh HCC tissues and adjacent nontumor tissues were collected to detect the mRNA levels of ONECUT2. 20 paired fresh metastatic and matched primary HCC tissues and adjacent nontumor tissues were collected to detect the protein levels of ONECUT2.

Patients underwent a regular check-up every 2–3 months in the first 2 years and every 3–6 months thereafter. The examinations of serum AFP level and abdominal ultrasonography were used for the evaluation of tumor recurrence. CT and/or MRI examinations were performed every 3–6 months, along with chest radiographic examination. Follow-up data were summarized at the end of December 2013 (Cohort I) and December 2016 (Cohort II) respectively. The primary endpoints were the overall survival and probability of recurrence. The period from surgery to recurrence was defined as the recurrence time. The period between surgery and death or last follow-up was defined as the overall survival time. The study was approved by the Ethics Committee of Tongji hospital of Tongji Medical College, with written informed consent from all patients.

### Immunohistochemistry

Liver tissues fixed in 10% formalin were embedded in paraffin, then were sliced 4-μm-thick sections. After drying in the oven at 60 °C for 1 h, and liver slices were deparaffinized with xylene and rehydrated with ethanol of gradient concentration. Endogenous peroxidase activity was quenched with 3% H_2_O_2_ for 12 min. Then, the sections were incubated with primary antibodies ONECUT2 (Atlas Antibodies, HPA057058, 1:250), ACLY (Atlas Antibodies, HPA022434, 1:3000) and FGF2 (Abcam, ab92337, 1:2000) at 4 °C overnight. The next day, a peroxidase-conjugated second antibody (Santa Cruz) was added to incubate the sections for 30 min at room temperature. The signals were visualized following diaminobenzidine treatment for 2 min. A light microscope (Olympus, Japan) was used to capture the images.

Two independent pathologists performed an analysis of the IHC results. The staining intensity was scored: negative, 0; weak, 1; medium, 2; strong, 3. The percentage of positive cells was scored: negative, 0; 1–25%, 1; 26–50%, 2; 51–75%, 3; 76–100%, 4. The above two scores were multiplied to determine the final immunoreactivity scores, yielding a range from 0 to 12. Scores ranging from 0 to 3 were considered “negative”, and scores ranging from 4 to 12 were considered “positive”.

### The human Cancer PathwayFinder RT^2^ profiler PCR array

The human Cancer PathwayFinder RT^2^ profiler PCR array was conducted in PLC/PRF/5-ONECUT2 vs. PLC/PRF/5-control and SNU398-ONECUT2 vs. SNU398-control. The cDNA was synthesized using the RT^2^ First Strand Kit (Qiagen). The cDNA synthesis reaction was mixed with 2× RT^2^ qPCR SYBR Green Mastermix and ddH_2_O, and then dispensed to the PCR array 96-well plate (25 μL/well). The Bio-Rad CFX96 was used to perform a 2-step cycling program. Data normalization was done by correcting all Ct values based on the average Ct values of several housekeeping genes present on array.

### Plasmid construction

The complete CDS construct of ONECUT2, pCMV-ONECUT2, was generated by using forward and reverse primers incorporating EcoRI and XhoI sites at the 5' and 3'-ends and then cloned into the EcoRI and XhoI sites of the pCMV-Tag2B vector. Besides, the FGF2 promoter sequence (−2888/+108) was obtained from human genomic DNA using PCR. This sequence is located at the position of the transcriptional start site (−2888 to +108) in the 5′-flanking region of the human FGF2 gene. The vector was constructed by incorporating forward and reverse primers at the 5′- and 3′-ends of the SacI and XhoI sites, respectively. The PCR product was cloned into the SacI and XhoI sites of the pGL3-basic vector (Promega). Similarly, constructs containing a deletion of the 5’-flanking region of the FGF2 promoter, (−2577/+108)FGF2, (−2228/+108)FGF2, (−1439/+108)FGF2, (−992/+108)FGF2, were generated based on the (−2888/+108)FGF2 construct as the template. The QuikChange II Site-Directed Mutagenesis Kit (Stratagene) was used to mutate the binding sites of ONECUT2 to FGF2 promoter. Other promoter constructs were generated similarly. The sequence integrity of all constructs was verified by DNA sequencing. All primers were provided in Supplementary Table S7.

### Lentivirus transfection

Lentiviral vectors containing small hairpin RNA (shRNA) targeting ONECUT2, ACLY, FGF2, ELK1, FGFR1, FGFR2, FGFR3, and FGFR4 were generated using PLKO.1-TRC (Addgene). The vector “pLKO.1-puro Non-Target shRNA Control Plasmid DNA” (purchased from Sigma, SHC016) was used as the negative control. Lentiviral vectors encoding the human ONECUT2, ACLY and FGF2 genes were constructed in pLV-puro or pLV-neo (Addgene). For the generation of targeted lentivirus, HEK-293T cells were transfected with the recombinant vectors and packaging vectors pMD2G and psPAX2 (Addgene plasmid #12259 and #12260) using Lipofectamine^®^3000 (Thermo Fisher Scientific). After 48 h, viral supernatant was harvested by centrifugation and filtered with a 0.45-μm filter (Millipore, MA, USA). HCC cells were incubated with lentiviruses and 5 µg/ml polybrene (Sigma H9268). After transfection for 72 h, puromycin (2.5 μg/ml) was used to select stably transfected cells. The shRNA sequences were shown in Supplementary Table S8.

### Quantitative real-time PCR

Total RNA from HCC cells and samples was extracted using a RNeasy Plus Mini Kit (50) kit (Qiagen, Hilden, Germany) and then was reverse-transcribed using an Advantage RT-for-PCR Kit (Qiagen). RT-PCR was performed to amplify the target sequence using a SYBR Green PCR Kit (Qiagen). The relative mRNA expression was determined using the 2^–ΔΔCt^ method and normalized to the control groups. The primers used for qRT-PCR were listed in Supplementary Table S7.

### Western blotting

Protein from tissues and cells was subjected to SDS-PAGE electrophoresis and transferred onto nitrocellulose membranes. After blocking nonspecific binding sites with 5% BSA for 2 h, nitrocellulose membranes were incubated with primary antibodies overnight at 4 °C. The primary antibodies used are as follows: anti-ONECUT2 (1/500; 21916-1-AP, Proteintech, USA); anti-FGF2 (1/3000; ab92337, Abcam, USA); anti-ACLY (0.2 μg/ml; HPA022434, Atlas Antibodies, Sweden); anti-ELK1 (1/1000; 9182, Cell Signaling Technology, USA); anti-p-ELK1(Ser383) (1/1000; 9186, Cell Signaling Technology, USA); anti-p-Akt(Ser-473) (1/2000; 4060, Cell Signaling Technology, USA); anti-Akt(pan) (1/1000; 4685, Cell Signaling Technology, USA); anti-ERK, anti-P38, anti-JNK (1/1000; 9926, Cell signaling technology, USA); anti-p-ERK, anti-p-P38, anti-p-JNK (1/1000; 9910, Cell signaling technology, USA); anti-AMPKα (1/1000; 5831, Cell signaling technology, USA); anti-p-AMPKα (Thr172) (1/1000; 2535, Cell signaling technology, USA); anti-p-PKCα (T638) (1/5000; ab32502, Abcam, USA); anti-PKCα (1/1000; 2056, Cell Signaling Technology, USA); anti-p-FGFR1(Tyr653/654) (1/1000; 52928, Cell Signaling Technology, USA); anti-FGFR1 (1/1000; 9740, Cell Signaling Technology, USA); anti-FGFR2 (1/1000; ab109372, Abcam, USA); anti-FGFR3 (1/1000; ab133644, Abcam, USA); anti-FGFR4 (0.2 µg/ml; MAB6852, R&D, USA); anti-GAPDH (1/2000; 5174, Cell Signaling Technology, USA). The next day, an HRP-conjugated secondary antibody was added for incubation. ImmobilonTM Western Chemiluminescent HRP substrate (Millipore, USA) was used to visualize immunoreactive proteins.

### Enzyme-linked immunosorbent assay (ELISA)

FGF2 levels in cell culture supernatant were detected using an ELISA kit (R&D Systems, USA). A blank well was used for the zero setting. Absorbance values at 450 nm were read using a Synergy2 microplate reader (BioTek, USA). The experiment was conducted in triplicate, and the mean value was calculated.

### Luciferase reporter assay

1 × 10^5^ cells were plated in 24-well plates for 12–24 h. A mixture of 0.18 μg of promoter reporter plasmids, 0.02 μg of pRL-TK plasmids and 0.6 μg of expression vector plasmids were cotransfected into cells using Lipofectamine 3000 (Invitrogen, USA). After 48 h, cells transfected with luciferase reporter constructs were lysed and then centrifuged for 3 min. Luciferase activity was measured using the Dual-Luciferase Assay (Promega, USA). The transfection efficiencies were normalized by cotransfection with the Renilla luciferase expression plasmid pRL-SV40 (Promega, USA). The relative luciferase activity was measured using a Modulus^TM^ TD20/20 Luminometer (Turner Biosystems, USA).

### Chromatin immunoprecipitation assay (ChIP)

HCC and normal liver tissues were first cut into small pieces (≤1 mm^3^). Then, DNase I (20 mg/mL; Sigma-Aldrich) and collagenase (1.5 mg/mL; Sigma-Aldrich) were added for digestion. The 1× red cell lysis was used for red blood cell. 1% formaldehyde was used to crosslink dissociated cells for 10 min. Sonication was used to fragment DNA from cell lysate. The fragmented DNA was immunoprecipitated when incubating with ChIP grade antibody anti-ONECUT2 (Proteintech, 21916-1-AP) and anti-ELK1 (Abcam, ab32106) at 4 °C. Then, specific primers were designed (provided in Supplementary Table S7) and RT-PCR was performed for amplification of the corresponding binding fragments on the promoters.

### Colony formation assay

1 × 10^3^ cells were plated in six-well plates. After routine culture for 14 days, cells formed stable colonies. The cell colonies were fixed with 4% paraformaldehyde, followed by stained with crystal violet solution. The positive colonies (>50 cells/colony) were determined under a microscope (Olympus, Japan).

### CCK-8 assay

1 × 10^3^ cells were plated in 96-well plates. The cell proliferation rate was detected using CCK-8 solution (Beyotime, Jiangsu, China) after culturing for 0–4 days. Absorbance values at 450 nm were measured using a Synergy2 microplate reader (BioTek, USA) and all samples were measured three times.

### Transwell assays

For migration assays, 4 × 10^4^ HCC cells were resuspended in serum-free medium and then seeded onto the upper chamber of 8-µm pore membranes (Corning, USA). Complete medium containing 10% FBS (Gibco, Australia) as a chemoattractant was added to the lower chamber. Twenty-four hours later, the cells located on the lower chamber were fixed with 4% paraformaldehyde, stained with 0.2% crystal violet and photographed using inverted fluorescence microscope. Five fields per membrane were randomly selected and then the average number was determined. For invasion assays, 100 μl of Matrigel (200 mg/ml) were added to the upper chamber at 37 °C for 6 h, followed by the implant of 8 × 10^4^ HCC cells. Other experimental procedures were performed as previously described. Triplicate assays were used for each experiment.

### HCC orthotopic mouse model

BALB/C nude mice aged 5-week were maintained and treated under ethical guidelines approved by the Committee on the Use of Live Animals in Teaching and Research, Tongji Medical College. In anaesthetized mice (ten per group), 50 μl mixture with 2 × 10^6^ indicated PLC/PRF/5 cells or MHCC97H cells and Matrigel was orthotopically transplanted into left liver lobe through an 8 mm incision in epigastrium. The bioluminescence was used to monitor the tumor growth of mice liver in vivo. Following intraperitoneal injection with 100 mg/kg D-Luciferin (Perkin-Elmer), bioluminescent signals were acquired by Lago X optical imaging system Imaging System (SI Imaging). A routine record was performed for the survival of nude mice. Nine weeks after orthotopic implantation, nude mice were humanely euthanized, and their lungs were harvested for the detection of metastatic foci by haematoxylin and eosin (H&E) staining.

### Statistical analysis

SPSS software (version 21.0) was used for statistical analysis. Data were presented as mean ± SD. Student’s *t*-test was used for quantitative data. Fisher’s exact test was used for categorical data. *P* < 0.05 was considered significant. Survival curves of HCC patients were plotted by the Kaplan–Meier method. The independent factors for survival and recurrence were determined by multivariate Cox proportional hazards regression models.

## Supplementary information

Supplementary Materials

Related MS file

Related MS file

## References

[1] Bray F, Ferlay J, Soerjomataram I, Siegel RL, Torre LA, Jemal A (2018). Global cancer statistics 2018: GLOBOCAN estimates of incidence and mortality worldwide for 36 cancers in 185 countries. CA Cancer J Clin.

[2] Bruix J, Reig M, Sherman M (2016). Evidence-based diagnosis, staging, and treatment of patients with hepatocellular carcinoma. Gastroenterology.

[3] Xu L, Hui L, Wang S, Gong J, Jin Y, Wang Y (2001). Expression profiling suggested a regulatory role of liver-enriched transcription factors in human hepatocellular carcinoma. Cancer Res.

[4] Jacquemin P, Lannoy VJ, Rousseau GG, Lemaigre FP (1999). OC-2, a novel mammalian member of the ONECUT class of homeodomain transcription factors whose function in liver partially overlaps with that of hepatocyte nuclear factor-6. J Biol Chem.

[5] Margagliotti S, Clotman F, Pierreux CE, Beaudry JB, Jacquemin P, Rousseau GG (2007). The Onecut transcription factors HNF-6/OC-1 and OC-2 regulate early liver expansion by controlling hepatoblast migration. Dev Biol.

[6] Rankovic B, Zidar N, Zlajpah M, Bostjancic E. Epithelial-mesenchymal transition-related microRNAs and their target genes in colorectal cancerogenesis. J Clin Med. 2019;8. 10.3390/jcm8101603 PMC6832722 31623346

[7] Wang GH, Zhou YM, Yu Z, Deng JP, Liu SF, Wei CZ (2020). Up-regulated ONECUT2 and down-regulated SST promote gastric cell migration, invasion, epithelial-mesenchymal transition and tumor growth in gastric cancer. Eur Rev Med Pharm Sci.

[8] Jiang Z, Tai Q, Xie X, Hou Z, Liu W, Yu Z (2021). EIF4A3-induced circ_0084615 contributes to the progression of colorectal cancer via miR-599/ONECUT2 pathway. J Exp Clin Cancer Res.

[9] Wu B, Zhang L, Yu Y, Lu T, Zhang Y, Zhu W (2020). miR-6086 inhibits ovarian cancer angiogenesis by downregulating the OC2/VEGFA/EGFL6 axis. Cell Death Dis.

[10] Chen J, Chen J, Sun B, Wu J, Du C (2020). ONECUT2 accelerates tumor proliferation through activating ROCK1 expression in gastric cancer. Cancer Manag Res.

[11] Zhang J, Cheng J, Zeng Z, Wang Y, Li X, Xie Q (2015). Comprehensive profiling of novel microRNA-9 targets and a tumor suppressor role of microRNA-9 via targeting IGF2BP1 in hepatocellular carcinoma. Oncotarget.

[12] Wang Y, Liu D, Zhang T, Xia L. FGF/FGFR signaling in hepatocellular carcinoma: from carcinogenesis to recent therapeutic intervention. Cancers. 2021;13. 10.3390/cancers13061360 PMC8002748 33802841

[13] Schulze-Osthoff K, Risau W, Vollmer E, Sorg C (1990). In situ detection of basic fibroblast growth factor by highly specific antibodies. Am J Pathol.

[14] Gospodarowicz D, Neufeld G, Schweigerer L. Fibroblast growth factor: structural and biological properties. J Cell Physiol Suppl. 1987;Suppl 5:15–26 10.1002/jcp.1041330405 2824530

[15] Jin-no K, Tanimizu M, Hyodo I, Kurimoto F, Yamashita T (1997). Plasma level of basic fibroblast growth factor increases with progression of chronic liver disease. J Gastroenterol.

[16] Poon RT, Ng IO, Lau C, Yu WC, Fan ST, Wong J (2001). Correlation of serum basic fibroblast growth factor levels with clinicopathologic features and postoperative recurrence in hepatocellular carcinoma. Am J Surg.

[17] Menendez JA, Lupu R (2007). Fatty acid synthase and the lipogenic phenotype in cancer pathogenesis. Nat Rev Cancer.

[18] Rohrig F, Schulze A (2016). The multifaceted roles of fatty acid synthesis in cancer. Nat Rev Cancer.

[19] Watson JA, Fang M, Lowenstein JM (1969). Tricarballylate and hydroxycitrate: substrate and inhibitor of ATP: citrate oxaloacetate lyase. Arch Biochem Biophys.

[20] Zaidi N, Swinnen JV, Smans K (2012). ATP-citrate lyase: a key player in cancer metabolism. Cancer Res.

[21] Hatzivassiliou G, Zhao F, Bauer DE, Andreadis C, Shaw AN, Dhanak D (2005). ATP citrate lyase inhibition can suppress tumor cell growth. Cancer Cell.

[22] Wen J, Min X, Shen M, Hua Q, Han Y, Zhao L (2019). ACLY facilitates colon cancer cell metastasis by CTNNB1. J Exp Clin Cancer Res.

[23] Sato R, Okamoto A, Inoue J, Miyamoto W, Sakai Y, Emoto N (2000). Transcriptional regulation of the ATP citrate-lyase gene by sterol regulatory element-binding proteins. J Biol Chem.

[24] Migita T, Narita T, Nomura K, Miyagi E, Inazuka F, Matsuura M (2008). ATP citrate lyase: activation and therapeutic implications in non-small cell lung cancer. Cancer Res.

[25] Lin R, Tao R, Gao X, Li T, Zhou X, Guan KL (2013). Acetylation stabilizes ATP-citrate lyase to promote lipid biosynthesis and tumor growth. Mol Cell.

[26] Zhang C, Liu J, Huang G, Zhao Y, Yue X, Wu H (2016). Cullin3-KLHL25 ubiquitin ligase targets ACLY for degradation to inhibit lipid synthesis and tumor progression. Genes Dev.

[27] Gu L, Zhu Y, Lin X, Lu B, Zhou X, Zhou F (2021). The IKKbeta-USP30-ACLY axis controls lipogenesis and tumorigenesis. Hepatology.

[28] Katoh M, Nakagama H (2014). FGF receptors: cancer biology and therapeutics. Med Res Rev.

[29] Kin M, Sata M, Ueno T, Torimura T, Inuzuka S, Tsuji R (1997). Basic fibroblast growth factor regulates proliferation and motility of human hepatoma cells by an autocrine mechanism. J Hepatol.

[30] Kelleher FC, O’Sullivan H, Smyth E, McDermott R, Viterbo A (2013). Fibroblast growth factor receptors, developmental corruption and malignant disease. Carcinogenesis.

[31] Akl MR, Nagpal P, Ayoub NM, Tai B, Prabhu SA, Capac CM (2016). Molecular and clinical significance of fibroblast growth factor 2 (FGF2 /bFGF) in malignancies of solid and hematological cancers for personalized therapies. Oncotarget.

[32] Sharpe R, Pearson A, Herrera-Abreu MT, Johnson D, Mackay A, Welti JC (2011). FGFR signaling promotes the growth of triple-negative and basal-like breast cancer cell lines both in vitro and in vivo. Clin Cancer Res.

[33] Filippov S, Pinkosky SL, Newton RS (2014). LDL-cholesterol reduction in patients with hypercholesterolemia by modulation of adenosine triphosphate-citrate lyase and adenosine monophosphate-activated protein kinase. Curr Opin Lipido.

[34] Costa RH, Kalinichenko VV, Holterman AX, Wang X (2003). Transcription factors in liver development, differentiation, and regeneration. Hepatology.

[35] Lambert M, Jambon S, Depauw S, David-Cordonnier MH. Targeting transcription factors for cancer treatment. Molecules. 2018;23. 10.3390/molecules23061479 PMC6100431 29921764

[36] Schrem H, Klempnauer J, Borlak J (2002). Liver-enriched transcription factors in liver function and development. Part I: the hepatocyte nuclear factor network and liver-specific gene expression. Pharmcol Rev.

[37] Lai JP, Chien JR, Moser DR, Staub JK, Aderca I, Montoya DP (2004). hSulf1 Sulfatase promotes apoptosis of hepatocellular cancer cells by decreasing heparin-binding growth factor signaling. Gastroenterology.

[38] Liu Z, Wang Y, Dou C, Xu M, Sun L, Wang L (2018). Hypoxia-induced up-regulation of VASP promotes invasiveness and metastasis of hepatocellular carcinoma. Theranostics.

[39] Wellen KE, Hatzivassiliou G, Sachdeva UM, Bui TV, Cross JR, Thompson CB (2009). ATP-citrate lyase links cellular metabolism to histone acetylation. Science.

[40] Lu M, Zhu WW, Wang X, Tang JJ, Zhang KL, Yu GY (2019). ACOT12-dependent alteration of acetyl-CoA drives hepatocellular carcinoma metastasis by epigenetic induction of epithelial-mesenchymal transition. Cell Metab.

[41] Jung J, Zheng M, Goldfarb M, Zaret KS (1999). Initiation of mammalian liver development from endoderm by fibroblast growth factors. Science.

[42] Ogasawara S, Yano H, Iemura A, Hisaka T, Kojiro M (1996). Expressions of basic fibroblast growth factor and its receptors and their relationship to proliferation of human hepatocellular carcinoma cell lines. Hepatology.

[43] Fidler IJ (2003). The pathogenesis of cancer metastasis: the ‘seed and soil’ hypothesis revisited. Nat Rev Cancer.

[44] Pang R, Poon RT (2006). Angiogenesis and antiangiogenic therapy in hepatocellular carcinoma. Cancer Lett.

[45] Luo X, Cheng C, Tan Z, Li N, Tang M, Yang L (2017). Emerging roles of lipid metabolism in cancer metastasis. Mol Cancer.

[46] Chambers AF, Groom AC, MacDonald IC (2002). Dissemination and growth of cancer cells in metastatic sites. Nat Rev Cancer.

[47] Iwamoto H, Abe M, Yang Y, Cui D, Seki T, Nakamura M (2018). Cancer lipid metabolism confers antiangiogenic drug resistance. Cell Metab.

[48] Mohammadi M, Froum S, Hamby JM, Schroeder MC, Panek RL, Lu GH (1998). Crystal structure of an angiogenesis inhibitor bound to the FGF receptor tyrosine kinase domain. EMBO J.

[49] Montesdeoca N, Lopez M, Ariza X, Herrero L, Makowski K (2020). Inhibitors of lipogenic enzymes as a potential therapy against cancer. FASEB J.

