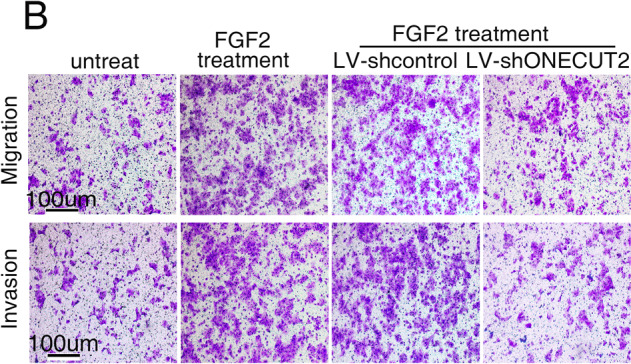# Correction to: ONECUT2 facilitates hepatocellular carcinoma metastasis by transcriptionally upregulating FGF2 and ACLY

**DOI:** 10.1038/s41419-021-04475-0

**Published:** 2021-12-23

**Authors:** Danfei Liu, Tongyue Zhang, Xiaoping Chen, Bixiang Zhang, Yijun Wang, Meng Xie, Xiaoyu Ji, Mengyu Sun, Wenjie Huang, Limin Xia

**Affiliations:** 1 Department of Gastroenterology, Institute of Liver and Gastrointestinal Diseases, Hubei Key Laboratory of Hepato-Pancreato-Biliary Diseases, Tongji Hospital of Tongji Medical College, Huazhong University of Science and Technology, Wuhan, 430030 Hubei China; 2 Hubei Key Laboratory of Hepato-Pancreato-Biliary Diseases; Hepatic Surgery Center, Tongji Hospital, Tongji Medical College, Huazhong University of Science and Technology; Clinical Medicine Research Center for Hepatic Surgery of Hubei Province; Key Laboratory of Organ Transplantation, Ministry of Education and Ministry of Public Health, Wuhan, Hubei 430030 China

**Keywords:** Liver cancer, Metastasis

Correction to: *Cell Death & Disease* 10.1038/s41419-021-04410-3, published online 27 November 2021

The original version of this article unfortunately contained a mistake. There was an error in figure 6b. The authors apologize for the error. The corrected figure can be found below. The original article has been corrected.