# Supplementary material for: ONECUT2 facilitates hepatocellular carcinoma metastasis by transcriptionally upregulating FGF2 and ACLY
Source: Cell Death Dis. 2021 Nov 27;12(12):1113. doi: 10.1038/s41419-021-04410-3 (PMC8627506; doi:10.1038/s41419-021-04410-3)
Supplement: Supplementary file 1 — Supplementary Materials [file 41419_2021_4410_MOESM1_ESM.docx]

**Supplementary materials**

**Supplementary Figure S1** **ONECUT2 is significantly elevated in HCC and facilitates cell proliferation and metastasis**


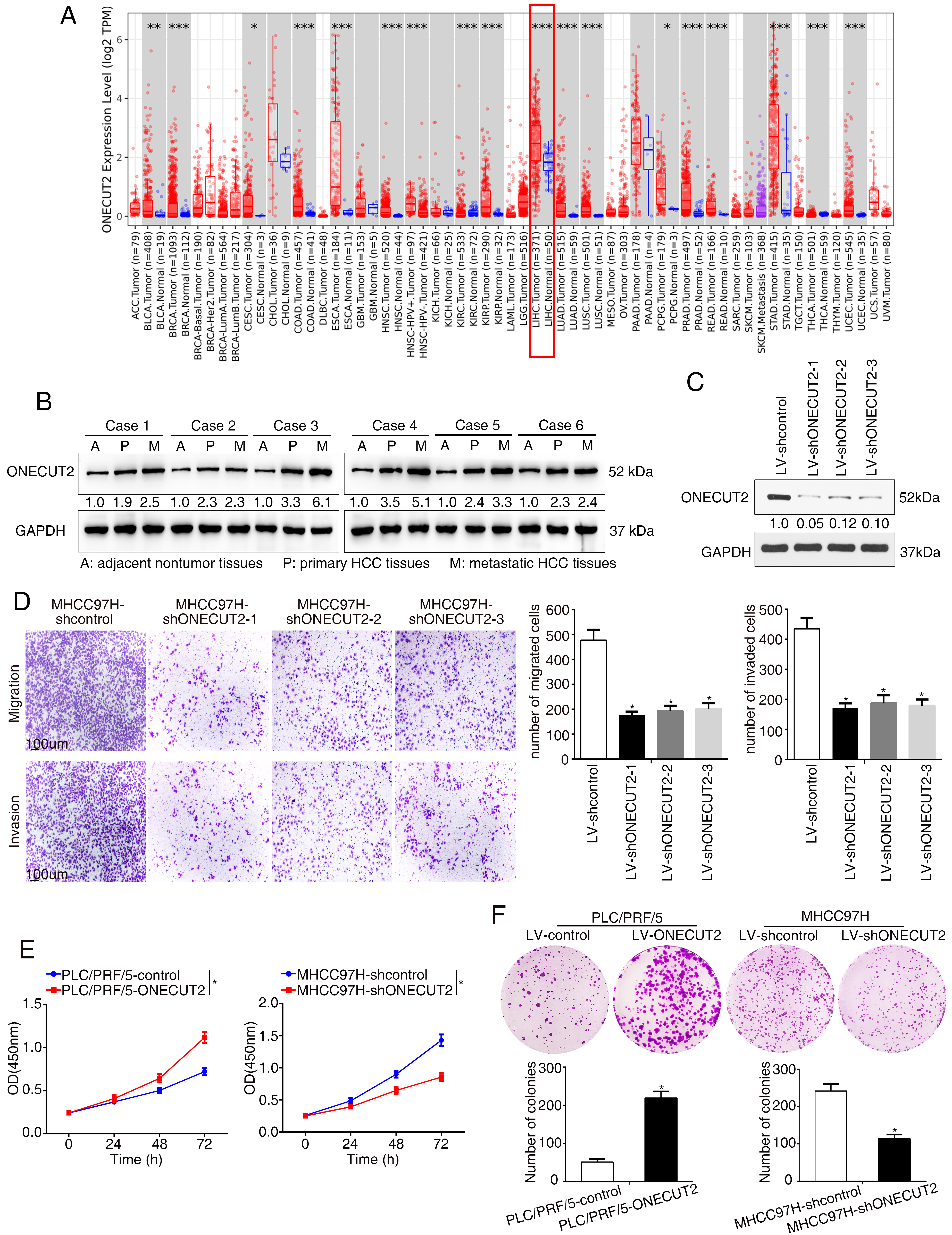


(A) The expression of ONECUT2 was analysed from TCGA database using TIMER website.

(B) Western blot analysis showing the protein levels of ONECUT2 in 6 pairs of fresh metastatic and matched primary HCC tissues and adjacent nontumor tissues.

(C) Western blot analysis showing the expression levels of ONECUT2 when MHCC97H cells were transfected with ONECUT2-silencing lentivirus.

(D) Transwell assays showing the migration and invasion of MHCC97H cells after ONECUT2-silencing lentivirus transfection.

(E-F) CCK8 and colony formation assays assessed the effect of ONECUT2 on HCC cell proliferation.

All the data are shown as mean±SD. *P<0.05.

**Supplementary Figure S2**

**
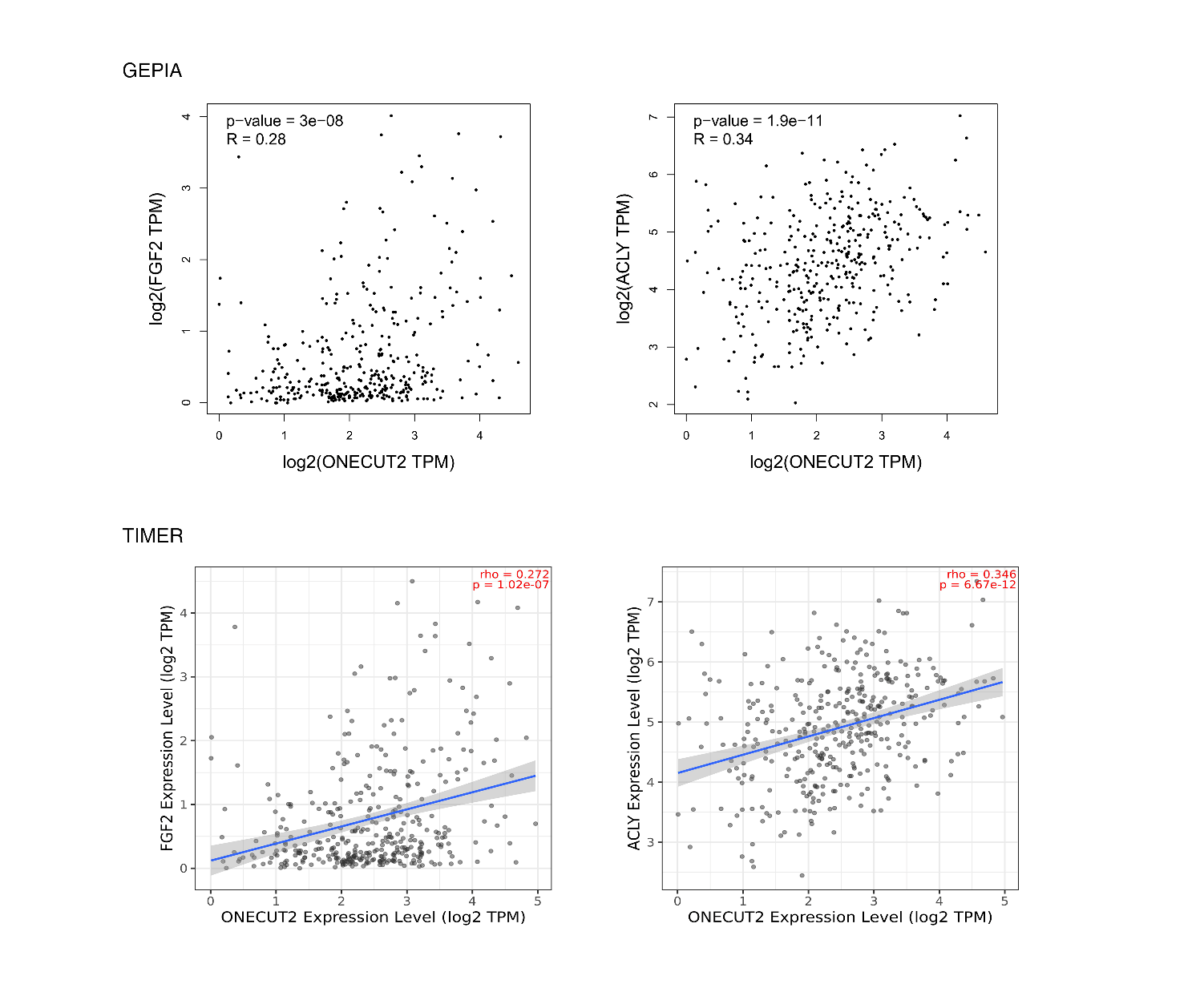
**

The correlation between ONECUT2 expression and FGF2 and ACLY expression was analysed by GEPIA and TIMER website.

**Supplementary Figure S3**

**
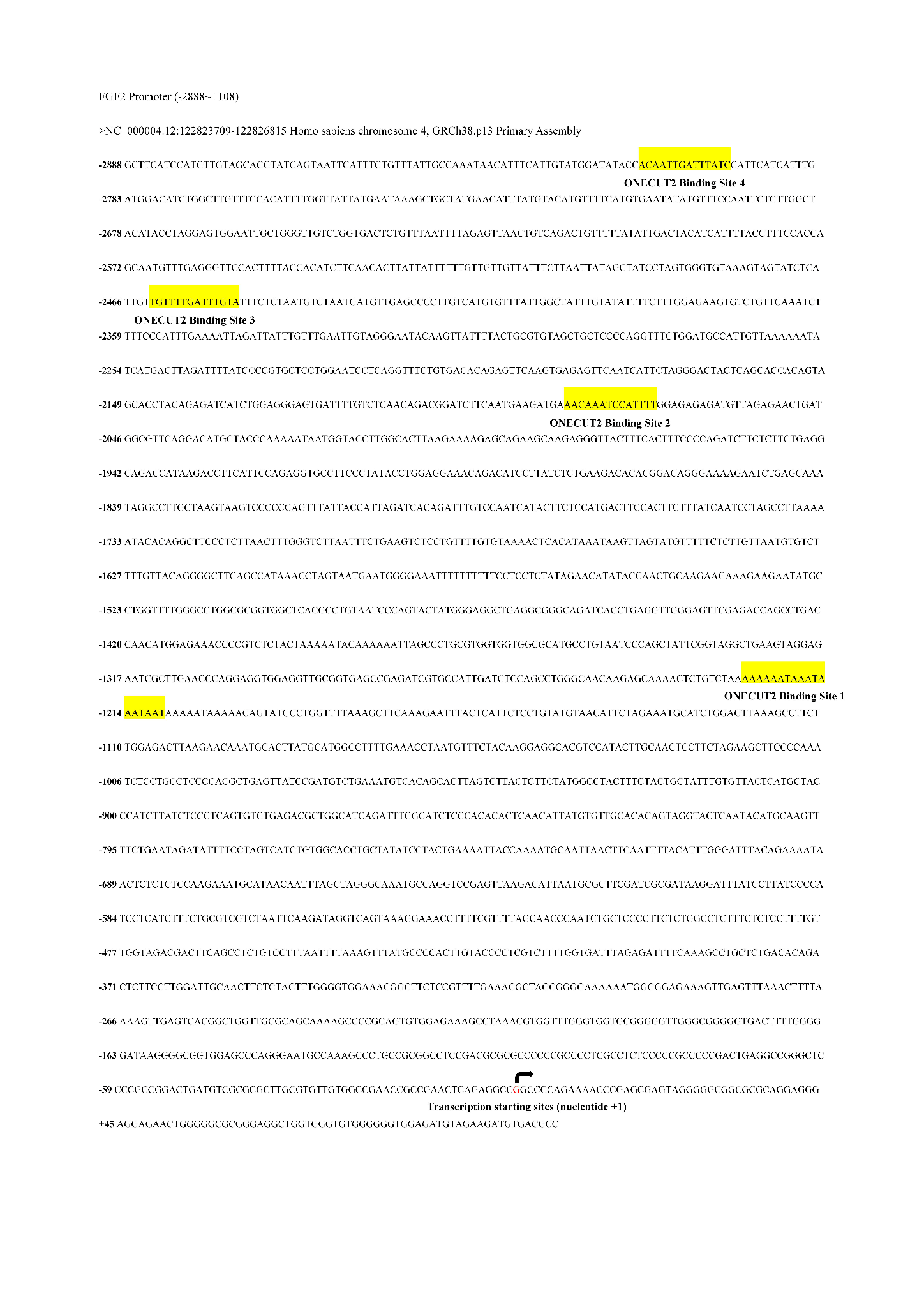
**

Sequence analysis showing the ONECUT2 binding motifs on FGF2 promoter.

**Supplementary Figure S4**

**
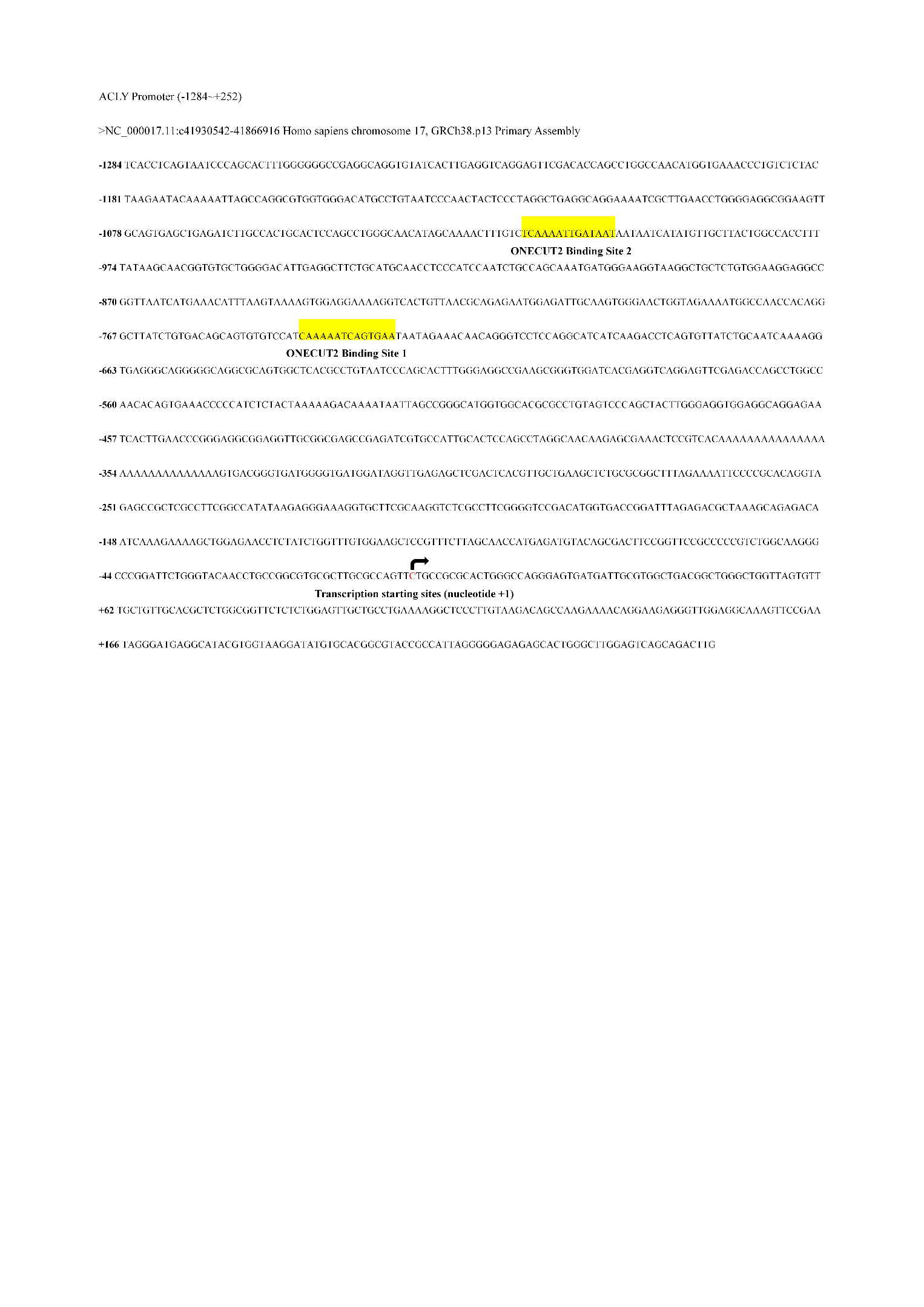
**

Sequence analysis showing the ONECUT2 binding motifs on ACLY promoter.

**Supplementary Figure S5**

**
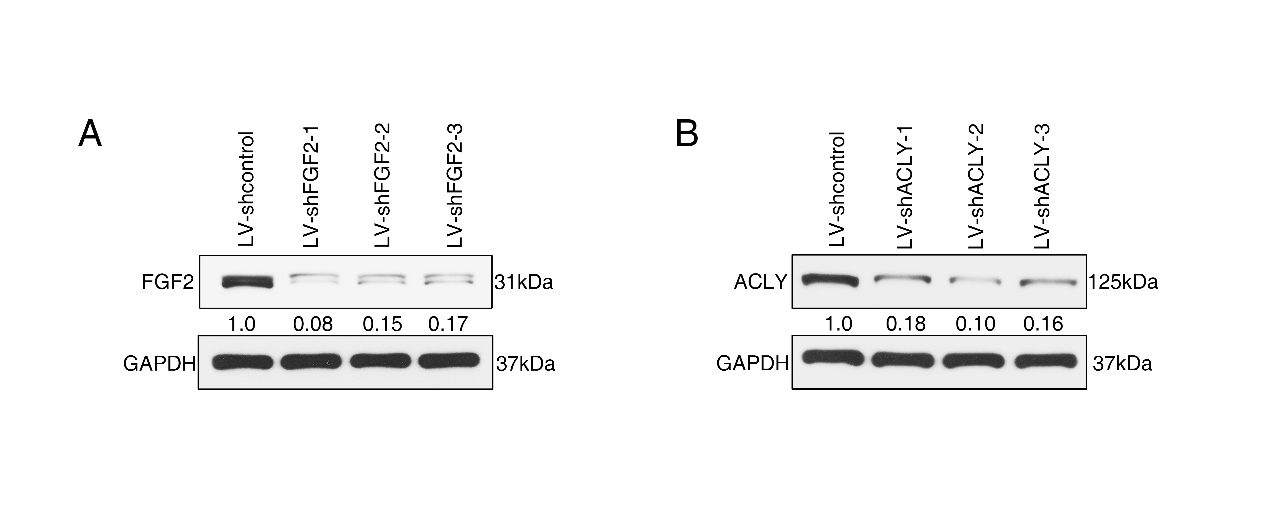
**(A-B) Western blot analysis showing the expression levels of FGF2 and ACLY when ONECUT2-overexpressing PLC/PRF/5 cells were transfected with FGF2-silencing or ACLY-silencing lentivirus.

**Supplementary Figure S6**

**
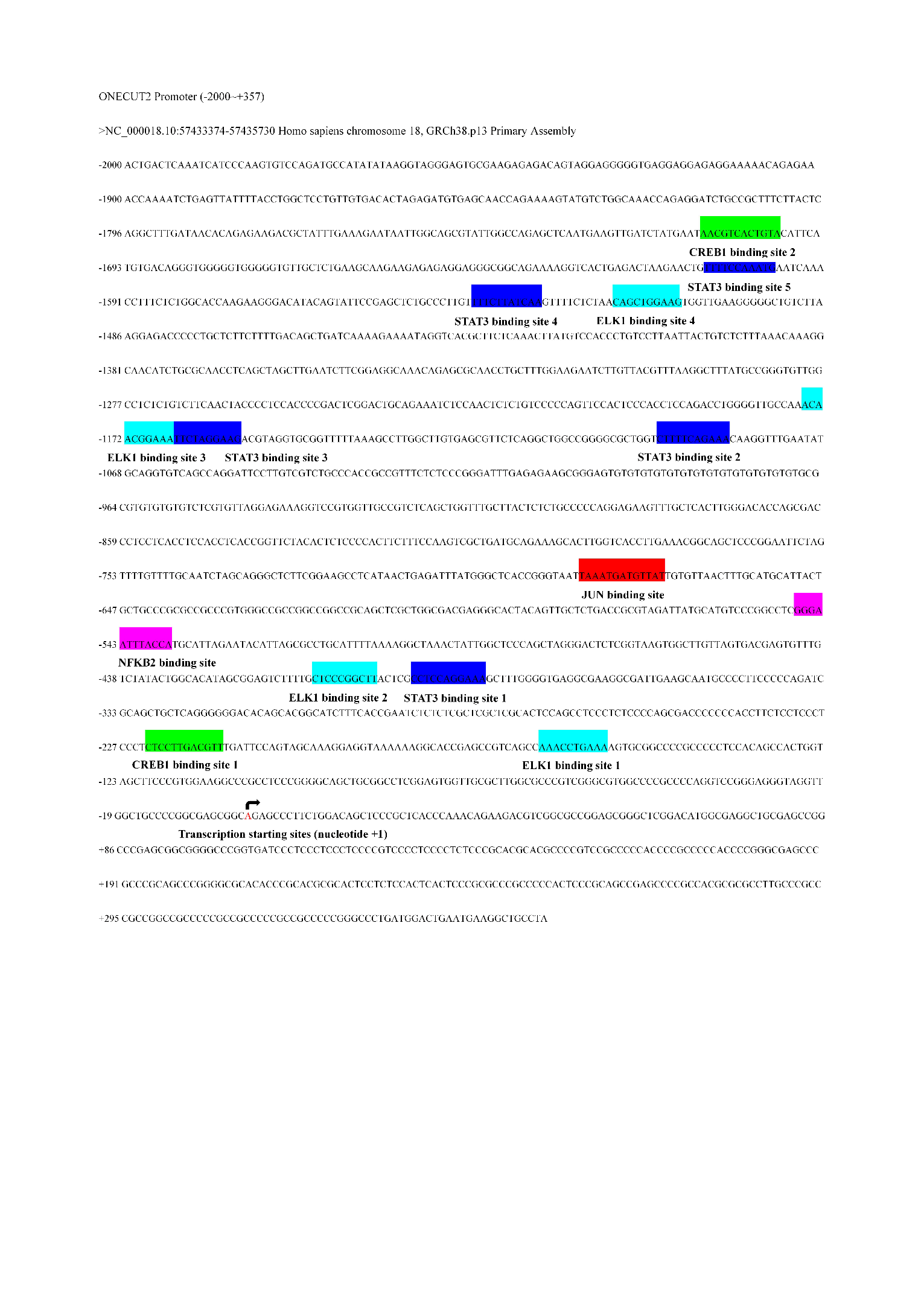
**

Sequence analysis showing the potential transcription factor binding sites on the promoter of ONECUT2.

**Supplementary Figure S7 ONECUT2 is essential for FGF2-facilitated cell proliferation, migration and invasion**

**
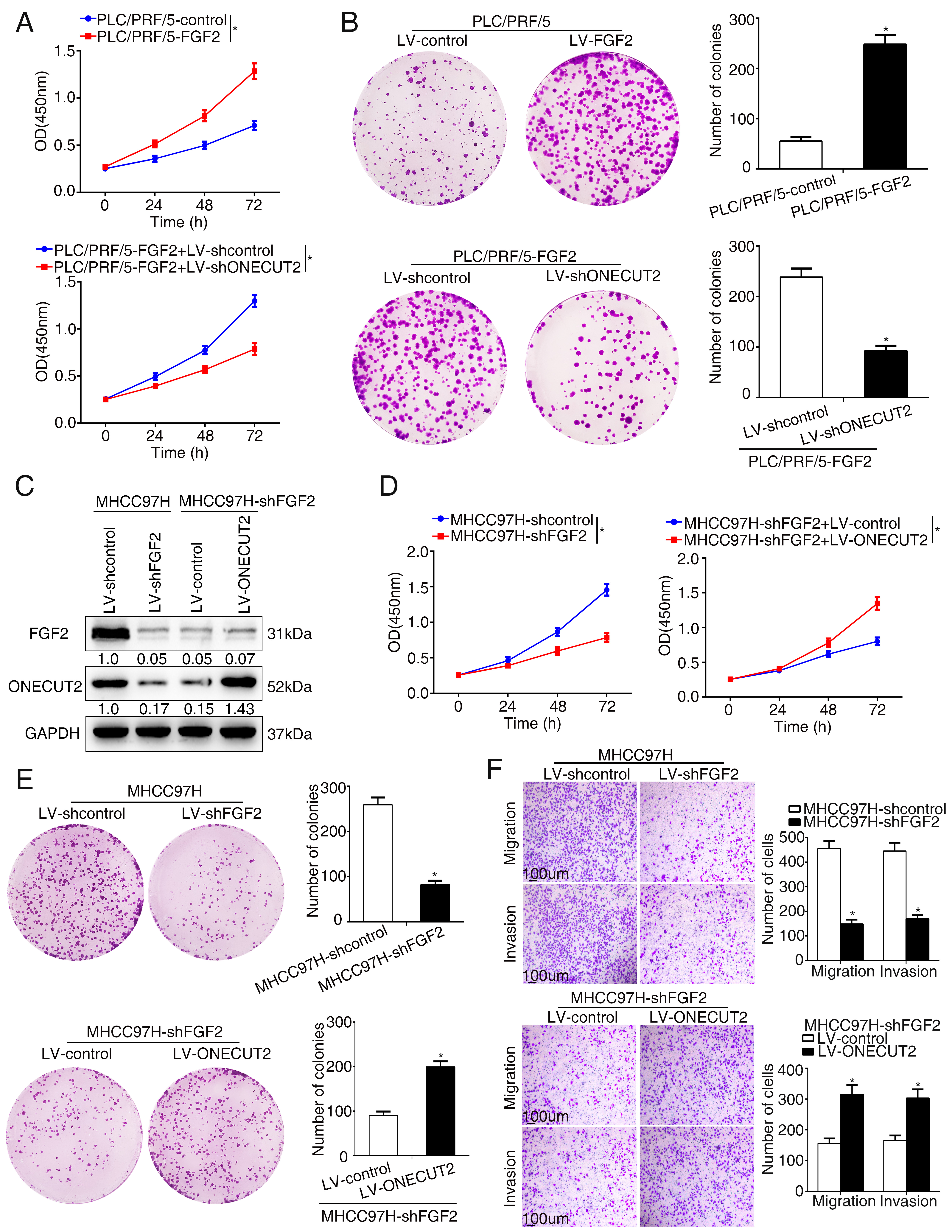
**

(A-B) CCK8 and colony formation assays showing the proliferation of PLC/PRF/5 cells after FGF2 overexpression and ONECUT2 knockdown.

(C) Western blot analysis showing the expression levels of FGF2 and ONECUT2 in MHCC97H cells after FGF2 knockdown and ONECUT2 overexpression.

(D-E) CCK8 and colony formation assays showing the proliferation of MHCC97H cells after FGF2 knockdown and ONECUT2 overexpression.

1. Transwell assays showing the migration and invasion of MHCC97H cells after FGF2 knockdown and ONECUT2 overexpression.

**Supplementary Figure S8 ACLY is responsible for FGF2-facilitated cell proliferation, migration and invasion**

**
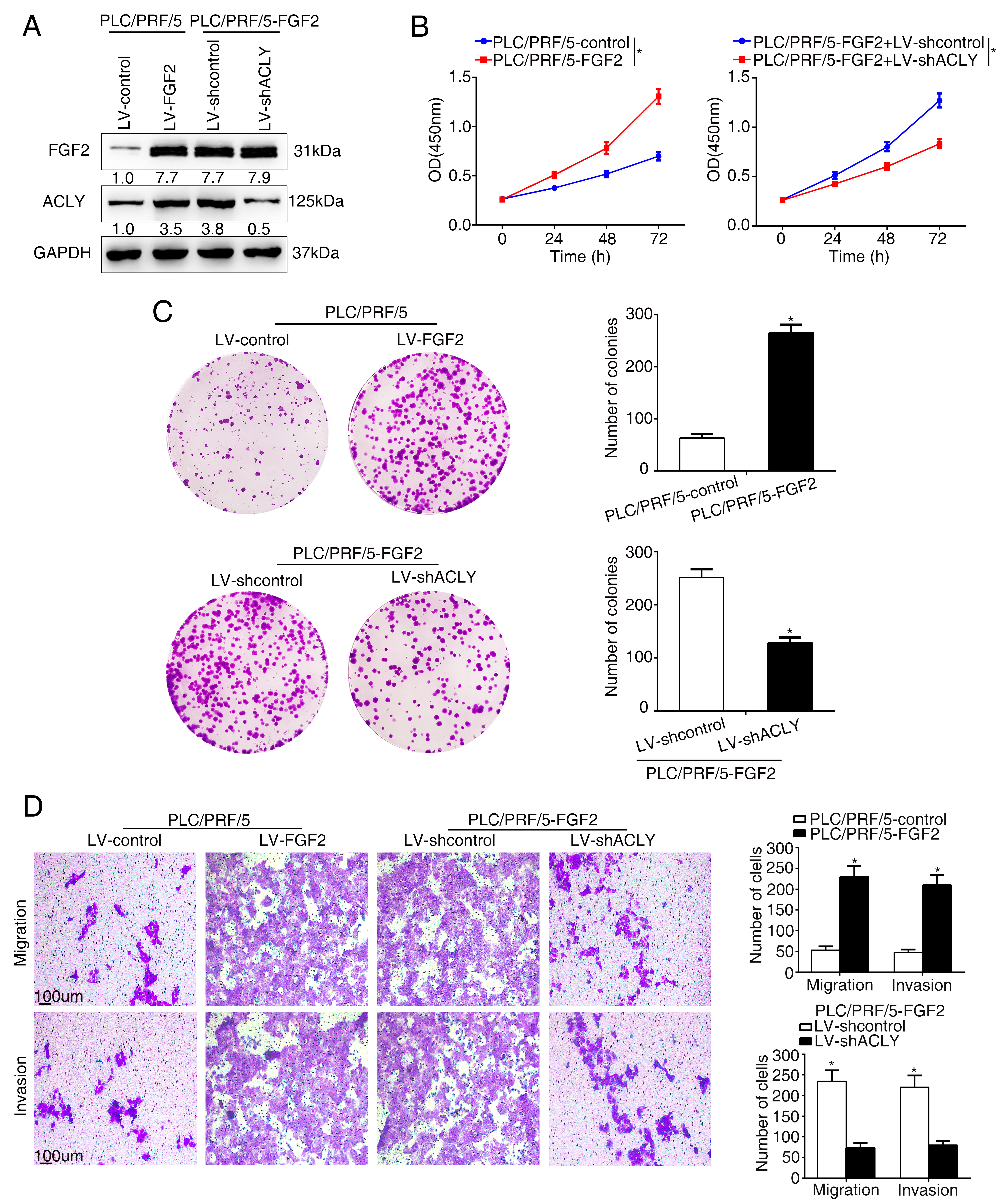
**

(A) Western blot analysis showing the expression levels of FGF2 and ACLY in PLC/PRF/5 cells after FGF2 overexpression and ACLY knockdown.

(B-C) CCK8 and colony formation assays showing the proliferation of PLC/PRF/5 cells after FGF2 overexpression and ACLY knockdown.

(D) Transwell assays showing the migration and invasion of PLC/PRF/5 cells after FGF2 overexpression and ACLY knockdown.

Supplementary Table S1 Univariate and Multivariate Analysis of Factors Associated with Time To Recurrence and Overall Survival in Cohort I HCC Patients (n=286)

|  | Time To Recurrence | |  | Overall Survival | |
| --- | --- | --- | --- | --- | --- |
| Clinical Variables | HR (95%CI) | P value |  | HR (95%CI) | P value |
| **Univariate analysis** |  |  |  |  |  |
| Age | 1.009(0.994-1.025) | 0.221 |  | 1.004(0.989-1.020) | 0.596 |
| Sex (female versus male) | 1.200(0.849-1.696) | 0.301 |  | 1.258(0.883-1.794) | 0.204 |
| Serum AFP (≤20 versus >20 ng/ml) | 0.786(0.591-1.046) | 0.098 |  | 0.836(0.626-1.116) | 0.225 |
| HBV infection (no versus yes) | 1.108(0.830-1.478) | 0.487 |  | 1.171(0.873-1.572) | 0.292 |
| Cirrhosis (absent versus present) | 1.144(0.868-1.509) | 0.339 |  | 1.121(0.845-1.487) | 0.430 |
| Child-pugh score (A versus B) | 0.916(0.623-1.345) | 0.654 |  | 0.968(0.650-1.440) | 0.871 |
| Tumor number (single versus multiple) | 0.632(0.486-0.823) | <0.001 |  | 0.404(0.307-0.531) | <0.001 |
| Maximal tumor size (≤5 versus >5 cm) | 0.643(0.494-0.838) | 0.001 |  | 0.661(0.505-0.866) | 0.003 |
| Tumor encapsulation (absent versus present) | 1.599(1.212-2.110) | 0.001 |  | 1.550(1.169-2.055) | 0.002 |
| Microvascular invasion (absent versus present) | 0.495(0.380-0.646) | <0.001 |  | 0.428(0.326-0.562) | <0.001 |
| Tumor differentiation (I-II versus III-Ⅳ) | 0.291(0.220-0.384) | <0.001 |  | 0.295(0.222-0.390) | <0.001 |
| TNM stage (I-II versus III) | 0.186(0.138-0.252) | <0.001 |  | 0.186(0.137-0.253) | <0.001 |
| ONECUT2 (negative versus positive) | 0.584(0.445-0.766) | <0.001 |  | 0.554(0.425-0.723) | <0.001 |
| **Multivariate analysis** |  |  |  |  |  |
| Tumor number (single versus multiple) | 0.893(0.675-1.183) | 0.431 |  | 0.440(0.331-0.584) | <0.001 |
| Maximal tumor size (≤5 versus >5 cm) | 1.092(0.820-1.454) | 0.546 |  | 1.074(0.805-1.432) | 0.630 |
| Tumor encapsulation (absent versus present) | 1.031(0.750-1.418) | 0.851 |  | 0.877(0.633-1.216) | 0.432 |
| Microvascular invasion (absent versus present) | 0.731(0.535-0.998) | 0.048 |  | 0.527(0.382-0.726) | <0.001 |
| Tumor differentiation (I-II versus III-Ⅳ) | 0.553(0.379-0.808) | 0.002 |  | 0.601(0.411-0.880) | 0.009 |
| TNM stage (I-II versus III) | 0.311(0.208-0.464) | <0.001 |  | 0.318(0.215-0.469) | <0.001 |
| ONECUT2 (negative versus positive) | 0.582(0.440-0.770) | <0.001 |  | 0.674(0.508-0.895) | 0.006 |

Supplementary Table S2 Univariate and Multivariate Analysis of Factors Associated with Time To Recurrence and Overall Survival in Cohort II HCC Patients (n=180)

|  | Time To Recurrence | |  | Overall Survival | |
| --- | --- | --- | --- | --- | --- |
| Clinical Variables | HR (95%CI) | P value |  | HR (95%CI) | P value |
| **Univariate analysis** |  |  |  |  |  |
| Age | 0.991(0.975-1.007) | 0.268 |  | 0.989(0.973-1.005) | 0.182 |
| Sex (female versus male) | 0.717(0.455-1.130) | 0.152 |  | 0.746(0.473-1.178) | 0.209 |
| Serum AFP (≤20 versus >20 ng/ml) | 1.267(0.868-1.849) | 0.220 |  | 1.304(0.889-1.914) | 0.175 |
| HBV infection (no versus yes) | 1.044(0.721-1.512) | 0.820 |  | 1.027(0.703-1.502) | 0.890 |
| Cirrhosis (absent versus present) | 1.206(0.840-1.732) | 0.311 |  | 1.173(0.812-1.694) | 0.394 |
| Child-pugh score (A versus B) | 0.988(0.670-1.458) | 0.953 |  | 0.952(0.644-1.407) | 0.804 |
| Tumor number (single versus multiple) | 0.234(0.160-0.340) | <0.001 |  | 0.212(0.144-0.313) | <0.001 |
| Maximal tumor size (≤5 versus >5 cm) | 0.651(0.467-0.907) | 0.011 |  | 0.608(0.433-0.856) | 0.004 |
| Tumor encapsulation (absent versus present) | 2.689(1.926-3.755) | <0.001 |  | 2.902(2.065-4.080) | <0.001 |
| Microvascular invasion (absent versus present) | 0.247(0.174-0.350) | <0.001 |  | 0.237(0.166-0.337) | <0.001 |
| Tumor differentiation (I-II versus III-Ⅳ) | 0.271(0.191-0.385) | <0.001 |  | 0.261(0.183-0.373) | <0.001 |
| TNM stage (I-II versus III) | 0.148(0.097-0.225) | <0.001 |  | 0.130(0.085-0.199) | <0.001 |
| ONECUT2 (negative versus positive) | 0.355(0.252-0.500) | <0.001 |  | 0.314(0.221-0.447) | <0.001 |
| **Multivariate analysis** |  |  |  |  |  |
| Tumor number (single versus multiple) | 0.274(0.181-0.414) | <0.001 |  | 0.246(0.161-0.376) | <0.001 |
| Maximal tumor size (≤5 versus >5 cm) | 0.736(0.519-1.043) | 0.085 |  | 0.746(0.492-1.012) | 0.058 |
| Tumor encapsulation (absent versus present) | 0.679(0.420-1.099) | 0.115 |  | 0.714(0.441-1.156) | 0.170 |
| Microvascular invasion (absent versus present) | 0.441(0.261-0.746) | 0.002 |  | 0.444(0.261-0.756) | 0.003 |
| Tumor differentiation (I-II versus III-Ⅳ) | 0.623(0.393-0.988) | 0.044 |  | 0.602(0.384-0.943) | 0.027 |
| TNM stage (I-II versus III) | 0.257(0.155-0.424) | <0.001 |  | 0.234(0.141-0.388) | <0.001 |
| ONECUT2 (negative versus positive) | 0.548(0.370-0.812) | 0.003 |  | 0.494(0.330-0.740) | 0.001 |

Supplementary Table S3. List of genes differentially expressed in PLC/PRF/5-ONECUT2 versus PLC/PRF/5-control cells using Human Cancer PathwayFinder PCR array

| **gene** | **PLC/PRF/5-ONECUT2**  **vs**  **PLC/PRF/5-control** | **Description** |
| --- | --- | --- |
| FGF2 | 5.68 | Fibroblast growth factor 2 (basic) |
| ACLY | 5.02 | ATP citrate lyase |
| VEGFA | 4.13 | Vascular endothelial growth factor A |
| ACSL4 | 3.37 | Acyl-CoA synthetase long-chain family member 4 |
| CDH2 | 3.21 | Cadherin 2, type 1, N-cadherin (neuronal) |
| MCM2 | 3.04 | Minichromosome maintenance complex component 2 |
| PGF | 3.01 | Placental growth factor |
| CDC20 | 2.71 | Cell division cycle 20 homolog (S. cerevisiae) |
| AURKA | 2.68 | Aurora kinase A |
| E2F4 | 2.66 | E2F transcription factor 4, p107/p130-binding |
| DSP | 2.63 | Desmoplakin |
| FLT4 | 2.54 | Fms-related tyrosine kinase 4 (vascular endothelial growth factor/vascular permeability factor receptor) |
| BIRC3 | 2.41 | Baculoviral IAP repeat containing 3 |
| ANGPT1 | 2.36 | Angiopoietin 1 |
| FASLG | 2.35 | Fas ligand (TNF superfamily, member 6) |
| KDR | 2.35 | Kinase insert domain receptor (a type III receptor tyrosine kinase) |
| ARNT | 2.34 | Aryl hydrocarbon receptor nuclear translocator |
| ADM | 2.31 | Adrenomedullin |
| ANGPT2 | 2.26 | Angiopoietin 2 |
| BMI1 | 2.17 | BMI1 polycomb ring finger oncogene |
| IGFBP5 | 2.16 | Insulin-like growth factor binding protein 5 |
| SNAI1 | 2.16 | Snail homolog 1 (Drosophila) |
| CCL2 | 2.13 | Chemokine (C-C motif) ligand 2 |
| LIG4 | 1.81 | Ligase IV, DNA, ATP-dependent |
| SNAI2 | 1.77 | Snail homolog 2 (Drosophila) |
| LPL | 1.68 | Lipoprotein lipase |
| MAP2K1 | 1.62 | Mitogen-activated protein kinase kinase 1 |
| SNAI3 | 1.65 | Snail homolog 3 (Drosophila) |
| CCND2 | 1.52 | Cyclin D2 |
| G6PD | 1.48 | Glucose-6-phosphate dehydrogenase |
| NOL3 | 1.37 | Nucleolar protein 3 (apoptosis repressor with CARD domain) |
| GSC | 1.36 | Goosecoid homeobox |
| ETS2 | 1.34 | V-Ets erythroblastosis virus E26 oncogene homolog 2 (avian) |
| LDHA | 1.32 | Lactate dehydrogenase A |
| XIAP | 1.31 | X-linked inhibitor of apoptosis |
| TERF2IP | 1.31 | Telomeric repeat binding factor 2, interacting protein |
| CA9 | 1.26 | Carbonic anhydrase IX |
| SOD1 | 1.25 | Superoxide dismutase 1, soluble |
| IGFBP3 | 1.25 | Insulin-like growth factor binding protein 3 |
| TNKS | 1.24 | Tankyrase, TRF1-interacting ankyrin-related ADP-ribose polymerase |
| SKP2 | 1.22 | S-phase kinase-associated protein 2 (p45) |
| KRT14 | 1.16 | Keratin 14 |
| DKC1 | 1.13 | Dyskeratosis congenita 1, dyskerin |
| PFKL | 1.08 | Phosphofructokinase, liver |
| SLC2A1 | 1.08 | Solute carrier family 2 (facilitated glucose transporter), member 1 |
| FOXC2 | 1.07 | Forkhead box C2 (MFH-1, mesenchyme forkhead 1) |
| EPO | 1.06 | Erythropoietin |
| SERPINB2 | 1.06 | Serpin peptidase inhibitor, clade B (ovalbumin), member 2 |
| SOX10 | 1.05 | SRY (sex determining region Y)-box 10 |
| POLB | 1.05 | Polymerase (DNA directed), beta |
| DDB2 | 1.04 | Damage-specific DNA binding protein 2, 48kDa |
| TERF1 | 1.03 | Telomeric repeat binding factor (NIMA-interacting) 1 |
| ERCC3 | 1.01 | Excision repair cross-complementing rodent repair deficiency, complementation group 3 (xeroderma pigmentosum group B complementing) |
| TBX2 | 1.01 | T-box 2 |
| PPP1R15A | -1.02 | Protein phosphatase 1, regulatory (inhibitor) subunit 15A |
| BCL2L11 | -1.06 | BCL2-like 11 (apoptosis facilitator) |
| TEP1 | -1.06 | Telomerase-associated protein 1 |
| CFLAR | -1.08 | CASP8 and FADD-like apoptosis regulator |
| IGFBP7 | -1.08 | Insulin-like growth factor binding protein 7 |
| CASP7 | -1.32 | Caspase 7, apoptosis-related cysteine peptidase |
| WEE1 | -1.33 | WEE1 homolog (S. pombe) |
| CCND3 | -1.34 | Cyclin D3 |
| COX5A | -1.36 | Cytochrome c oxidase subunit Va |
| TNKS2 | -1.36 | Tankyrase, TRF1-interacting ankyrin-related ADP-ribose polymerase 2 |
| DDIT3 | -1.39 | DNA-damage-inducible transcript 3 |
| TEK | -1.42 | TEK tyrosine kinase, endothelial |
| OCLN | -1.43 | Occludin |
| TINF2 | -1.45 | TERF1 (TRF1)-interacting nuclear factor 2 |
| ERCC5 | -1.46 | Excision repair cross-complementing rodent repair deficiency, complementation group 5 |
| MAPK14 | -1.46 | Mitogen-activated protein kinase 14 |
| STMN1 | -1.47 | Stathmin 1 |
| ATP5A1 | -1.54 | ATP synthase, H+ transporting, mitochondrial F1 complex, alpha subunit 1, cardiac muscle |
| SERPINF1 | -1.54 | Serpin peptidase inhibitor, clade F (alpha-2 antiplasmin, pigment epithelium derived factor), member 1 |
| MKI67 | -1.55 | Antigen identified by monoclonal antibody Ki-67 |
| GADD45G | -1.61 | Growth arrest and DNA-damage-inducible, gamma |
| MAP2K3 | -1.61 | Mitogen-activated protein kinase kinase 3 |
| CPT2 | -1.62 | Carnitine palmitoyltransferase 2 |
| HMOX1 | -1.67 | Heme oxygenase (decycling) 1 |
| UQCRFS1 | -1.82 | Ubiquinol-cytochrome c reductase, Rieske iron-sulfur polypeptide 1 |
| PINX1 | -2.06 | PIN2/TERF1 interacting, telomerase inhibitor 1 |
| CASP2 | -2.06 | Caspase 2, apoptosis-related cysteine peptidase |
| APAF1 | -2.36 | Apoptotic peptidase activating factor 1 |
| GPD2 | -2.63 | Glycerol-3-phosphate dehydrogenase 2 (mitochondrial) |
| CASP9 | -2.81 | Caspase 9, apoptosis-related cysteine peptidase |

Supplementary Table S4. List of genes differentially expressed in SNU398-ONECUT2 versus SNU398-control cells using Human Cancer PathwayFinder PCR array

| gene | **SNU398-ONECUT2**  **vs**  **SNU398-control** | Description |
| --- | --- | --- |
| ACLY | 5.13 | ATP citrate lyase |
| FGF2 | 4.72 | Fibroblast growth factor 2 (basic) |
| VEGFA | 3.44 | Vascular endothelial growth factor A |
| KDR | 3.23 | Kinase insert domain receptor (a type III receptor tyrosine kinase) |
| G6PD | 3.02 | Glucose-6-phosphate dehydrogenase |
| ACSL4 | 2.77 | Acyl-CoA synthetase long-chain family member 4 |
| ARNT | 2.75 | Aryl hydrocarbon receptor nuclear translocator |
| IGFBP3 | 2.61 | Insulin-like growth factor binding protein 3 |
| FLT4 | 2.55 | Fms-related tyrosine kinase 4 (vascular endothelial growth factor/vascular permeability factor receptor) |
| SNAI1 | 2.46 | Snail homolog 1 (Drosophila) |
| TERF1 | 2.37 | Telomeric repeat binding factor (NIMA-interacting) 1 |
| MAP2K1 | 2.36 | Mitogen-activated protein kinase kinase 1 |
| ANGPT2 | 2.33 | Angiopoietin 2 |
| SNAI3 | 2.24 | Snail homolog 3 (Drosophila) |
| FOXC2 | 2.21 | Forkhead box C2 (MFH-1, mesenchyme forkhead 1) |
| ETS2 | 2.16 | V-Ets erythroblastosis virus E26 oncogene homolog 2 (avian) |
| ANGPT1 | 2.14 | Angiopoietin 1 |
| BMI1 | 1.92 | BMI1 polycomb ring finger oncogene |
| IGFBP5 | 1.85 | Insulin-like growth factor binding protein 5 |
| SNAI2 | 1.72 | Snail homolog 2 (Drosophila) |
| FASLG | 1.83 | Fas ligand (TNF superfamily, member 6) |
| PGF | 1.76 | Placental growth factor |
| BIRC3 | 1.73 | Baculoviral IAP repeat containing 3 |
| CDC20 | 1.71 | Cell division cycle 20 homolog (S. cerevisiae) |
| CCL2 | 1.71 | Chemokine (C-C motif) ligand 2 |
| DSP | 1.68 | Desmoplakin |
| LIG4 | 1.63 | Ligase IV, DNA, ATP-dependent |
| CDH2 | 1.58 | Cadherin 2, type 1, N-cadherin (neuronal) |
| SKP2 | 1.57 | S-phase kinase-associated protein 2 (p45) |
| LPL | 1.49 | Lipoprotein lipase |
| XIAP | 1.46 | X-linked inhibitor of apoptosis |
| SOD1 | 1.44 | Superoxide dismutase 1, soluble |
| CCND2 | 1.42 | Cyclin D2 |
| CA9 | 1.41 | Carbonic anhydrase IX |
| MCM2 | 1.36 | Minichromosome maintenance complex component 2 |
| SLC2A1 | 1.36 | Solute carrier family 2 (facilitated glucose transporter), member 1 |
| ADM | 1.34 | Adrenomedullin |
| KRT14 | 1.34 | Keratin 14 |
| AURKA | 1.28 | Aurora kinase A |
| DDB2 | 1.28 | Damage-specific DNA binding protein 2, 48kDa |
| DKC1 | 1.25 | Dyskeratosis congenita 1, dyskerin |
| LDHA | 1.24 | Lactate dehydrogenase A |
| POLB | 1.22 | Polymerase (DNA directed), beta |
| SERPINB2 | 1.21 | Serpin peptidase inhibitor, clade B (ovalbumin), member 2 |
| NOL3 | 1.16 | Nucleolar protein 3 (apoptosis repressor with CARD domain) |
| SOX10 | 1.16 | SRY (sex determining region Y)-box 10 |
| E2F4 | 1.13 | E2F transcription factor 4, p107/p130-binding |
| EPO | 1.11 | Erythropoietin |
| GSC | 1.08 | Goosecoid homeobox |
| TERF2IP | 1.05 | Telomeric repeat binding factor 2, interacting protein |
| TNKS | 1.05 | Tankyrase, TRF1-interacting ankyrin-related ADP-ribose polymerase |
| WEE1 | 1.03 | WEE1 homolog (S. pombe) |
| ERCC3 | -1.01 | Excision repair cross-complementing rodent repair deficiency, complementation group 3 (xeroderma pigmentosum group B complementing) |
| TEK | -1.02 | TEK tyrosine kinase, endothelial |
| CCND3 | -1.06 | Cyclin D3 |
| COX5A | -1.08 | Cytochrome c oxidase subunit Va |
| PFKL | -1.13 | Phosphofructokinase, liver |
| TBX2 | -1.15 | T-box 2 |
| DDIT3 | -1.16 | DNA-damage-inducible transcript 3 |
| TINF2 | -1.25 | TERF1 (TRF1)-interacting nuclear factor 2 |
| TEP1 | -1.26 | Telomerase-associated protein 1 |
| ATP5A1 | -1.26 | ATP synthase, H+ transporting, mitochondrial F1 complex, alpha subunit 1, cardiac muscle |
| GADD45G | -1.31 | Growth arrest and DNA-damage-inducible, gamma |
| MAPK14 | -1.35 | Mitogen-activated protein kinase 14 |
| OCLN | -1.37 | Occludin |
| IGFBP7 | -1.38 | Insulin-like growth factor binding protein 7 |
| PPP1R15A | -1.42 | Protein phosphatase 1, regulatory (inhibitor) subunit 15A |
| CFLAR | -1.42 | CASP8 and FADD-like apoptosis regulator |
| BCL2L11 | -1.46 | BCL2-like 11 (apoptosis facilitator) |
| PINX1 | -1.46 | PIN2/TERF1 interacting, telomerase inhibitor 1 |
| TNKS2 | -1.52 | Tankyrase, TRF1-interacting ankyrin-related ADP-ribose polymerase 2 |
| ERCC5 | -1.52 | Excision repair cross-complementing rodent repair deficiency, complementation group 5 |
| UQCRFS1 | -1.55 | Ubiquinol-cytochrome c reductase, Rieske iron-sulfur polypeptide 1 |
| STMN1 | -1.58 | Stathmin 1 |
| SERPINF1 | -1.61 | Serpin peptidase inhibitor, clade F (alpha-2 antiplasmin, pigment epithelium derived factor), member 1 |
| CASP7 | -1.62 | Caspase 7, apoptosis-related cysteine peptidase |
| MKI67 | -1.63 | Antigen identified by monoclonal antibody Ki-67 |
| CPT2 | -2.01 | Carnitine palmitoyltransferase 2 |
| APAF1 | -2.05 | Apoptotic peptidase activating factor 1 |
| CASP9 | -2.33 | Caspase 9, apoptosis-related cysteine peptidase |
| MAP2K3 | -2.36 | Mitogen-activated protein kinase kinase 3 |
| GPD2 | -2.46 | Glycerol-3-phosphate dehydrogenase 2 (mitochondrial) |
| CASP2 | -2.61 | Caspase 2, apoptosis-related cysteine peptidase |
| HMOX1 | -2.67 | Heme oxygenase (decycling) 1 |

Supplementary Table S5 Correlation between FGF2 expression and clinicopathological characteristics in two independent cohorts of human HCC tissues

|  |  | Cohort I | |  |  | Cohort II | |  |
| --- | --- | --- | --- | --- | --- | --- | --- | --- |
| Clinicopathological variables | | Tumor FGF2 expression | | *P* Value |  | Tumor FGF2 expression | | *P* Value |
|  |  | Negative (n=134) | Positive (n=152) |  |  | Negative (n=83) | Positive (n=97) |  |
| Age | | 52.16(9.220) | 52.99(8.566) | 0.461 |  | 52.54(10.844) | 49.85(9.786) | 0.376 |
| Sex | female | 21 | 26 | 0.752 |  | 15 | 17 | 1.000 |
|  | male | 113 | 126 |  |  | 68 | 80 |  |
| Serum AFP | ≤20ng/ml | 41 | 54 | 0.382 |  | 22 | 20 | 0.381 |
|  | >20ng/ml | 93 | 98 |  |  | 61 | 77 |  |
| Virus infection | HBV | 89 | 115 | 0.318 |  | 57 | 76 | 0.439 |
|  | HCV | 16 | 13 |  |  | 10 | 7 |  |
|  | HBV+HCV | 7 | 8 |  |  | 3 | 4 |  |
|  | none | 22 | 16 |  |  | 13 | 10 |  |
| Cirrhosis | absent | 42 | 55 | 0.453 |  | 23 | 28 | 1.000 |
|  | present | 92 | 97 |  |  | 60 | 69 |  |
| Child-pugh score | Class A | 114 | 137 | 0.210 |  | 67 | 70 | 0.220 |
|  | Class B | 20 | 15 |  |  | 16 | 27 |  |
| Tumor number | single | 74 | 65 | 0.044* |  | 42 | 33 | 0.033* |
|  | multiple | 60 | 87 |  |  | 41 | 64 |  |
| Maximal tumor size | ≤5cm | 87 | 81 | 0.054 |  | 47 | 33 | 0.003* |
|  | >5cm | 47 | 71 |  |  | 36 | 64 |  |
| Tumor encapsulation | absent | 31 | 56 | 0.014* |  | 29 | 53 | 0.011* |
|  | present | 103 | 96 |  |  | 54 | 44 |  |
| Microvascular invasion | absent | 84 | 74 | 0.023* |  | 63 | 38 | <0.001* |
|  | present | 50 | 78 |  |  | 20 | 59 |  |
| Tumor differentiation | I-II | 106 | 88 | <0.001* |  | 63 | 53 | 0.003* |
|  | III-Ⅳ | 28 | 64 |  |  | 20 | 44 |  |
| TNM stage | I-II | 115 | 97 | <0.001* |  | 74 | 69 | 0.003* |
|  | III | 19 | 55 |  |  | 9 | 28 |  |

*Statistically significant

Supplementary Table S6 Correlation between ACLY expression and clinicopathological characteristics in two independent cohorts of human HCC tissues

|  |  | Cohort I | |  |  | Cohort II | |  |
| --- | --- | --- | --- | --- | --- | --- | --- | --- |
|  |  | Tumor ACLY expression | | *P* Value |  | Tumor ACLY expression | | *P* Value |
| Clinicopathological variables | | Negative (n=127) | Positive (n=159) |  |  | Negative (n=77) | Positive (n=103) |  |
| Age | | 52.91(8.532) | 52.35(9.154) | 0.954 |  | 51.43(10.687) | 50.83(10.129) | 0.739 |
| Sex | female | 20 | 27 | 0.873 |  | 13 | 19 | 0.846 |
|  | male | 107 | 132 |  |  | 64 | 84 |  |
| Serum AFP | ≤20ng/ml | 47 | 48 | 0.256 |  | 22 | 20 | 0.159 |
|  | >20ng/ml | 80 | 111 |  |  | 55 | 83 |  |
| Virus infection | HBV | 91 | 113 | 0.527 |  | 56 | 77 | 0.694 |
|  | HCV | 14 | 15 |  |  | 7 | 10 |  |
|  | HBV+HCV | 4 | 11 |  |  | 2 | 5 |  |
|  | none | 18 | 20 |  |  | 12 | 11 |  |
| Cirrrhosis | absent | 43 | 54 | 1.000 |  | 23 | 28 | 0.739 |
|  | present | 84 | 105 |  |  | 54 | 75 |  |
| Child-pugh score | Class A | 108 | 143 | 0.276 |  | 58 | 79 | 0.861 |
|  | Class B | 19 | 16 |  |  | 19 | 24 |  |
| Tumor number | single | 73 | 66 | 0.009* |  | 40 | 35 | 0.022* |
|  | multiple | 54 | 93 |  |  | 37 | 68 |  |
| Maximal tumor size | ≤5cm | 87 | 81 | 0.004* |  | 43 | 37 | 0.010* |
|  | >5cm | 40 | 78 |  |  | 34 | 66 |  |
| Tumor encapsulation | absent | 30 | 57 | 0.028* |  | 25 | 57 | 0.003* |
|  | present | 97 | 102 |  |  | 52 | 46 |  |
| Microvascular invasion | absent | 84 | 74 | 0.001* |  | 55 | 46 | <0.001* |
|  | present | 43 | 85 |  |  | 22 | 57 |  |
| Tumor differentiation | I-II | 102 | 92 | <0.001* |  | 59 | 57 | 0.004* |
|  | III-Ⅳ | 25 | 67 |  |  | 18 | 46 |  |
| TNM stage | I-II | 112 | 100 | <0.001* |  | 73 | 70 | <0.001* |
|  | III | 15 | 59 |  |  | 4 | 33 |  |

Supplementary Table S7. Primer sequences used in the study

| **Primer name** | **Primer sequences** | **Enzyme** |
| --- | --- | --- |
| Primers for real-time PCR: |  |  |
| ONECUT2 sense | 5’- GGAATCCAAAACCGTGGAGTAA -3’ |  |
| ONECUT2 antisense | 5’- CTCTTTGCGTTTGCACGCTG -3’ |  |
| ACLY sense: | 5’- ATCGGTTCAAGTATGCTCGGG -3’ |  |
| ACLY antisense: | 5’- GACCAAGTTTTCCACGACGTT -3’ |  |
| FGF2 sense | 5'- AGTGTGTGCTAACCGTTACCT -3' |  |
| FGF2 antisense | 5'- ACTGCCCAGTTCGTTTCAGTG -3' |  |
| GAPDH sense: | 5’-ACAACTTTGGTATCGTGGAAGG-3’ |  |
| GAPDH antisense: | 5’-GCCATCACGCCACAGTTTC-3’ |  |
| **Primers for ACLY promoter construct:** |  |  |
| (-1284/+252) ACLY sense: | 5'- TATAGCTAGCTCACCTCAGTAATCCCAG -3' | Nhel |
| (-895/+252) ACLY sense: | 5'- TATAGCTAGCTAAGGCTGCTCTGTGGAA -3' | Nhel |
| (-398/+252) ACLY sense: | 5'- TATAGCTAGCCCTAGGCAACAAGAGCGA -3' | Nhel |
| antisense: | 5'- ATATCTCGAGCAAGTCTGCTGACTCCAA -3' | XhoI |
| **Primers for ACLY promoter site-directed mutagenesis:** | |  |
| ONECUT2 binding site 1 mutation sense: | 5'- GTCCATCAAA**ggct**AGTGAATAATAG -3' |  |
| ONECUT2 binding site 1 mutation antisense: | 5'- CTATTATTCACTagccTTTGATGGAC -3' |  |
| ONECUT2 binding site 2 mutation sense: | 5'- TTTGTCTCAAA**gcct**ATAATAATAAT -3' |  |
| ONECUT2 binding site 2 mutation antisense: | 5'- ATTATTATTATaggcTTTGAGACAAA -3' |  |
| **Primers used for ChIP in the ACLY promoter:** | |  |
| distant region sense: | 5’-GAGATGATTACCATAGTC-3’ |  |
| distant region antisense: | 5’-GACTCGGAGATTGCAGTG-3’ |  |
| binding site 1 sense: | 5’-GCAGGAGAATCACTTGAACC-3’ |  |
| binding site 1 antisense: | 5’-TGAGTCGAGCTCTCAACCTA-3’ |  |
| **Primers for FGF2 promoter construct:** | |  |
| (-2888/+108) FGF2 sense: | 5’-TATAGAGCTCGCTTCATCCATGTTGTAG-3’ | Sac I |
| (-2577/+108) FGF2 sense: | 5’-TATAGAGCTCCACCAGCAATGTTTGAGG-3’ | Sac I |
| (-2228/+108) FGF2 sense: | 5’-TATAGAGCTCTCCTGGAATCCTCAGGTT-3’ | Sac I |
| (-1439/+108) FGF2 sense: | 5’-TATAGAGCTCAGTTCGAGACCAGCCTGA-3’ | Sac I |
| (-992/+108) FGF2 sense: | 5’-TATAGAGCTCACGCTGAGTTATCCGATG-3’ | Sac I |
| Antisense: | 5’-ATATCTCGAGGGCGTCACATCTTCTACA-3’ | Xho I |
| **Primers for FGF2 promoter site-directed mutagenesis:** | |  |
| ONECUT2 binding site 4 mutation sense: | 5’-TATACCACAA**cctg**TTTATCCATTCA-3’ |  |
| ONECUT2 binding site 4 mutation antisense: | 5’-TGAATGGATAAAcaggTTGTGGTATA-3’ |  |
| ONECUT2 binding site 3 mutation sense: | 5’-CATTGTTGTT**cctg**TTTGTATTTCTC-3’ |  |
| ONECUT2 binding site 3 mutation antisense: | 5’-GAGAAATACAAAcaggAACAACAATG-3’ |  |
| ONECUT2 binding site 2 mutation sense: | 5’-AGATGAAACA**ggct**CATTTTGGAGAG-3’ |  |
| ONECUT2 binding site 2 mutation antisense: | 5’-CTCTCCAAAATGagccTGTTTCATCT-3’ |  |
| ONECUT2 binding site 1 mutation sense: | 5’-GTCTAAAAAAAAT**gggc**AAATAATAAAAAT-3’ |  |
| ONECUT2 binding site 1 mutation antisense: | 5’-ATTTTTATTATTTgcccATTTTTTTTAGAC-3’ |  |
| **Primers used for ChIP in the FGF2 promoter:** | |  |
| distant region sense: | 5’-CATCCTTAATAGCACTGG-3’ |  |
| distant region antisense: | 5’-CCGGAATTAGATAGTGGT-3’ |  |
| binding site 1 sense: | 5’-GTAGGAGAATCGCTTGAACC-3’ |  |
| binding site 1 antisense: | 5’-GGCTTTAACTCCAGATGCAT-3’ |  |
| **Primers for ONECUT2 promoter construct:** | |  |
| (-1820/+357) ONECUT2 sense: | 5’-TATAAGATCTAGAGGATCTGCCGCTTTC-3’ | BglⅡ |
| (-1328/+357) ONECUT2 sense: | 5’-TATAAGATCTACCTGCTTTGGAAGAATC-3’ | BglⅡ |
| (-795/+357) ONECUT2 sense: | 5’-TATAAGATCTAAGCACTTGGTCACCTTG-3’ | BglⅡ |
| (-308/+357) ONECUT2 sense: | 5’-TATAAGATCTCGGCATCTTTCACCGAAT-3’ | BglⅡ |
| (-82/+357) ONECUT2 sense: | 5’-TATAAGATCTTCGGAGTGGTTGCGCTT-3’ | BglⅡ |
| antisense: | 5’-ATATCTCGAGTAGGCAGCCTTCATTCAG-3’ | XhoI |
| **Primers for ONECUT2 promoter site-directed mutagenesis:** | |  |
| CREB1 binding site mutation sense: | 5’-TCCCTCTCCggtcCGTTTGATT-3’ |  |
| CREB1 binding site mutation antisense: | 5’-AATCAAACGgaccGGAGAGGGA-3’ |  |
| ELK1 binding site mutation sense: | 5’-CAGCCAAAttcaAAAAGTGC-3’ |  |
| ELK1 binding site mutation antisense: | 5’- GCACTTTTtgaaTTTGGCTG-3’ |  |
| **Primers used for ChIP in the ONECUT2 promoter:** | |  |
| distant region sense: | 5’-AGGCTACCTTAGCACTGG-3’ |  |
| distant region antisense: | 5’-TATATGGCATCTGGACAC-3’ |  |
| binding site sense: | 5’-CCAGTAGCAAAGGAGGTA-3’ |  |
| binding site antisense: | 5’-CAAGCGCAACCACTCCGA-3’ |  |
| **Primers for pCMV-ONECUT2 construction:** |  |  |
| Sense: | 5’-TGTTGAATTCATGAAGGCTGCCTACACCGC-3’ | EcoRI |
| Antisense: | 5’-TTGTCTCGAGTCATGCTTTGGTACACGTGC-3’ | XhoI |

Supplementary Table S8. Knockdown shRNA sequences used in this study

| Gene | Sequence |
| --- | --- |
| ONECUT2 |  |
| shRNA-1 | CCGGGCCATGAACAACCTCTACAGTCTCGAGACTGTAGAGGTTGTTCATGGCTTTTT |
| shRNA-2 | CCGGCAACCTCTACAGTCCCTACAACTCGAGTTGTAGGGACTGTAGAGGTTGTTTTT |
| shRNA-3 | CCGGCGAACACTCTTCGCCATCTTCCTCGAGGAAGATGGCGAAGAGTGTTCGTTTTTG |
| ACLY |  |
| shRNA-1 | CCGGCCTATGACTATGCCAAGACTACTCGAGTAGTCTTGGCATAGTCATAGGTTTTTG |
| shRNA-2 | CCGGCGAGGACTTGTACTTCACCTACTCGAGTAGGTGAAGTACAAGTCCTCGTTTTTG |
| shRNA-3 | CCGGCGTGAGAGCAATTCGAGATTACTCGAGTAATCTCGAATTGCTCTCACGTTTTTG |
| FGF2 |  |
| shRNA-1 | CCGGTATAGCTCAGTTTGGATAATTCTCGAGAATTATCCAAACTGAGCTATATTTTTG |
| shRNA-2 | CCGGTGAACGATTGGAATCTAATAACTCGAGTTATTAGATTCCAATCGTTCATTTTTG |
| shRNA-3 | CCGGGAAGATTACTGGCTTCTAAATCTCGAGATTTAGAAGCCAGTAATCTTCTTTTTG |
| shFGFR1 | CCGGCCACAGAATTGGAGGCTACAACTCGAGTTGTAGCCTCCAATTCTGTGGTTTTTG |
| shFGFR2 | GTACCGGAGCCCTGTTTGATAGAGTATACTCGAGTATACTCTATCAAACAGGGCTTTTTTTG |
| shFGFR3 | CCGGTGAAAGACGATGCCACTGACACTCGAGTGTCAGTGGCATCGTCTTTCATTTTT |
| shFGFR4 | CCGGCCCTCGAATAGGCACAGTTACCTCGAGGTAACTGTGCCTATTCGAGGGTTTTTG |
| shELK1 | CCGGCCTGCTTCCTACGCATACATTCTCGAGAATGTATGCGTAGGAAGCAGGTTTTT |
