# Supplementary material for: ONECUT2 facilitates hepatocellular carcinoma metastasis by transcriptionally upregulating FGF2 and ACLY
Source: Cell Death Dis. 2021 Nov 27;12(12):1113. doi: 10.1038/s41419-021-04410-3 (PMC8627506; doi:10.1038/s41419-021-04410-3)
Supplement: Supplementary file 3 — Related MS file [file 41419_2021_4410_MOESM3_ESM.pdf]

DECLARATION OF CONTRIBUTIONS TO ARTICLE

ADMC

Manuscript Number: CDDIS-21-3104R

Journal Name: *Cell Death & Disease* (the 'Journal')

Proposed Title of the Contribution:

ONECUT2 Facilitates Hepatocellular Carcinoma Metastasis by Transcriptionally Upregulating FGF2 and ACLY (the 'Contribution)

Author(s):

Danfei Liu, Tongyue Zhang, Xiaoping Chen, Bixiang Zhang, Yijun Wang, Meng Xie, Xiaoyu Ji, Mengyu Sun, Wenjie Huang*, Limin Xia,* (the 'Authors')

For all *CDDis* articles, each person named as an author in the published version must be able to show he or she has contributed substantially to the article.

Authorship credit should be based on 1) substantial contributions to conception and design, acquisition of data, or analysis and interpretation of data; 2) drafting the article or revising it critically for important intellectual content; and 3) final approval of the version to be published. Authors should meet conditions 1, 2 and 3.

Any person who cannot be shown to have made a substantial contribution to the article cannot be listed as an author in the final version. The name of any person who is deemed to have made a minor contribution can, however, appear in the Acknowledgments section of the article.

Please complete the table below to indicate the contributions of all named authors to the manuscript.

Author Full Name:	Specification of Contribution to the Manuscript:
Danfei Liu	designed studies, performed experiments and wrote the paper
Tongyue Zhang	assisted in immunohistochemistry staining and animal experiments
Xiaoping Chen	gave assistance in collecting and providing metastatic HCC samples
Bixiang Zhang	gave assistance in collecting and providing metastatic HCC samples
Yijun Wang	assisted in immunohistochemistry staining and animal experiments
Meng Xie	gave assistance in collecting tissues samples
Xiaoyu Ji	gave assistance in collecting tissues samples
Mengyu Sun	gave assistance in collecting tissues samples
Wenjie Huang	gave advice in experimental design
Limin Xia	designed studies

Please complete the table below to indicate the contributions of all named authors to the figures.

Figure 1:

Xiaoping Chen, Bixiang Zhang and Wenjie Huang gave assistance in collecting and providing metastatic HCC samples. Tongyue Zhang and Yijun Wang assisted in immunohistochemistry staining and animal experiments. Meng Xie, Xiaoyu Ji and Mengyu Sun gave assistance in collecting tissues samples.

Figure 2:

Danfei Liu performed qPCR, western blotting, ELISA, luciferase reporter assay and ChIP.

Figure 3:

Tongyue Zhang and Yijun Wang assisted in immunohistochemistry staining.

Figure 4:

Tongyue Zhang and Yijun Wang assisted in animal experiments.

Figure 5:

Danfei Liu performed qPCR, western blotting, luciferase reporter assay and ChIP.

Figure 6:

Tongyue Zhang and Yijun Wang assisted in animal experiments.

Signed for and on behalf of the Author(s):	Print Name:	Date:
Limin Xia	Limin Xia	Nov 06, 2021
